# The *cabABC* Operon Essential for Biofilm and Rugose Colony Development in *Vibrio vulnificus*

**DOI:** 10.1371/journal.ppat.1005192

**Published:** 2015-09-25

**Authors:** Jin Hwan Park, Youmi Jo, Song Yee Jang, Haenaem Kwon, Yasuhiko Irie, Matthew R. Parsek, Myung Hee Kim, Sang Ho Choi

**Affiliations:** 1 National Research Laboratory of Molecular Microbiology and Toxicology, Department of Agricultural Biotechnology, and Center for Food Safety and Toxicology, Seoul National University, Seoul, South Korea; 2 Infection and Immunity Research Center, Korea Research Institute of Bioscience and Biotechnology, Daejeon, South Korea; 3 Department of Microbiology, University of Washington, Seattle, Washington, United States of America; Northwestern University, Feinberg School of Medicine, UNITED STATES

## Abstract

A transcriptome analysis identified *Vibrio vulnificus cabABC* genes which were preferentially expressed in biofilms. The *cabABC* genes were transcribed as a single operon. The *cabA* gene was induced by elevated 3′,5′-cyclic diguanylic acid (c-di-GMP) and encoded a calcium-binding protein CabA. Comparison of the biofilms produced by the *cabA* mutant and its parent strain JN111 in microtiter plates using crystal-violet staining demonstrated that CabA contributed to biofilm formation in a calcium-dependent manner under elevated c-di-GMP conditions. Genetic and biochemical analyses revealed that CabA was secreted to the cell exterior through functional CabB and CabC, distributed throughout the biofilm matrix, and produced as the biofilm matured. These results, together with the observation that CabA also contributes to the development of rugose colony morphology, indicated that CabA is a matrix-associated protein required for maturation, rather than adhesion involved in the initial attachment, of biofilms. Microscopic comparison of the structure of biofilms produced by JN111 and the *cabA* mutant demonstrated that CabA is an extracellular matrix component essential for the development of the mature biofilm structures in flow cells and on oyster shells. Exogenously providing purified CabA restored the biofilm- and rugose colony-forming abilities of the *cabA* mutant when calcium was available. Circular dichroism and size exclusion analyses revealed that calcium binding induces CabA conformational changes which may lead to multimerization. Extracellular complementation experiments revealed that CabA can assemble a functional matrix only when exopolysaccharides coexist. Consequently, the combined results suggested that CabA is a structural protein of the extracellular matrix and multimerizes to a conformation functional in building robust biofilms, which may render *V*. *vulnificus* to survive in hostile environments and reach a concentrated infective dose.

## Author Summary

Biofilms are specialized and highly differentiated three-dimensional communities of bacteria encased in an extracellular polymeric matrix (EPM), and the bacteria’s mechanisms to form biofilm are closely linked to their virulence. The EPM often consists of polysaccharides, proteins, nucleic acids, and lipids. Compared to extracellular polysaccharides, little is known about the protein components in the biofilm matrix of *Vibrio vulnificus*, a foodborne pathogen. In this study, we identified and characterized *cabABC* genes which were preferentially expressed in biofilms. CabA is a calcium-binding protein and is secreted through functional CabB and CabC. Our results indicated that CabA contributes to the development of biofilm and rugose colony morphology under elevated c-di-GMP conditions. CabA is an extracellular matrix protein crucial for the structural integrity of robust biofilm in flow cells and on oyster shells. Calcium binding induces conformational changes and multimerization of CabA that may render the protein functional to build a well-structured matrix. CabA can assemble a functional matrix extracellularly only when exopolysaccharides (EPS) coexist, indicating that both CabA and EPS are required for the scaffold of *V*. *vulnificus* biofilm matrix. This is the first report on a non-polysaccharide matrix component that is essential for the development of the *V*. *vulnificus* biofilm structure.

## Introduction

Biofilm formation provides bacteria with protection from antimicrobial agents and host immune defense systems during infection as well as from a variety of stresses in the environment [1,2]. Therefore, biofilms of pathogenic bacteria are considered as one of the most important causes for new outbreaks and account for 65% of bacterial infections in humans [3]. Biofilm formation can be divided into sequential developmental stages beginning from initial surface attachment of planktonic bacteria continuing to multicellular structures (microcolony), subsequent maturation to biofilms, and detachment (dispersal) of bacterial cells from mature biofilms [1,2,4]. Mature biofilms are specialized and highly differentiated three-dimensional communities of bacteria encased in an extracellular polymeric matrix (EPM), the framework contributing to the organization and maintenance of biofilm structure and stability [2]. The major components of the EPM are polysaccharides, proteins, nucleic acids, and lipids, which are distributed between the cells in a non-homogeneous pattern [5]. Changes in colony morphology frequently reflect variations in biofilm matrix production levels, as smooth colony morphology of bacterial cells can switch to rugose (or wrinkled) by producing increased levels of EPM [4].

Exopolysaccharides (EPS) are the most prevalent component of *Vibrio* EPM and the structure, composition, and function of the *Vibrio cholerae* exopolysaccharide (VPS) and regulatory networks involved in its production have been studied as a model to delineate the molecular basis of bacterial biofilm formation [4,6]. Like many other pathogenic bacteria, *Vibrio vulnificus*, a pathogenic marine bacterium, forms biofilms in natural ecosystems [7,8,9]. There are several lines of evidence supporting that *V*. *vulnificus* embed themselves in oyster tissues and form biofilms to colonize and to persist in oyster as the primary infection route of the pathogen [10,11]. Diverse extracellular polysaccharides are produced by *V*. *vulnificus* and their functions in the development of biofilms are well characterized [8,12,13,14]. Among them, lipopolysaccharide (LPS) is known to play a role in the initial adhesion of planktonic cells to the surfaces of assay tubes [12]. A capsular polysaccharide (CPS), which is more tightly associated with the cell envelope than EPS, is also produced after maturation of biofilms [14,15,16]. CPS contributes to colony opacity and is required for determining biofilm size by limiting continual growth of mature biofilms [14,17]. Many, at least three, types of EPS are thought to play essential roles in the development of robust structures of EPM and mature biofilms of *V*. *vulnificus* [7,8,13,18,19].

The structural and regulatory genes involved in the *V*. *vulnificus* EPS production and, in turn, biofilm formation have been extensively studied [7,8,13,18,19,20]. The structural genes required for EPS production are organized into three clusters, EPS-I, II, and III on chromosome [13]. The cluster EPS-I consists of the *rbd* genes which are highly homologous to the *syp* genes involved in synthesis of the symbiotic-related polysaccharides of *Vibrio fischeri* [4,8]. The EPS-I genes were preferentially expressed in the estuarine-like conditions as determined by an RNA sequencing analysis [21]. The cluster EPS-II contains the *brp* genes (also *wcr* genes) which are organized into *brpT* encoding a putative transcription regulator and the *brpABCDFHIJK* operon required for the synthesis and transport of EPS [7,8,18,19]. The *brpA* mutant lost the ability to produce EPS, the parent biofilm phenotype, and rugose colony morphology [19]. Mutational analyses demonstrated that the third EPS cluster, EPS-III, is also involved in the production of EPS and formation of biofilms, but the functions of the EPS-III genes have not yet been addressed in details [13]. The *rbd* genes are regulated by RbdG [8], and all three clusters are positively regulated by NtrC at the transcription level [13]. Expression of the *brp* genes and production of EPS are regulated by 3′,5′-cyclic diguanylic acid (c-di-GMP) [7,22]. The secondary messenger c-di-GMP is synthesized by diguanylate cyclases (DGCs) containing the GGDEF domain and degraded by c-di-GMP-specific phosphodiesterases (PDEs) containing the EAL (or HD-GYP) domain which are ubiquitous in bacteria (for a recent review, [23]).

However, compared to EPS, little is known about the non-EPS components of the *V*. *vulnificus* EPM. Although a type IV pilin required for the initial adherence to HEp-2 cells and biofilm formation has been reported [24], no matrix proteins essential for the mature biofilm structures have been previously identified. In this study, a transcriptomic comparison of planktonic and biofilm cells of *V*. *vulnificus* led us to identify three genes, named *cabABC*, which are among the genes that are preferentially expressed in biofilms. Genetic, biochemical, and microscopic analyses were performed to demonstrate that CabA is a calcium-binding protein, produced as the biofilm matures, and secreted into the medium of the extracellular matrix through functional CabB and CabC. Construction of the *cabA* mutant and evaluation of its phenotypes provided evidences that CabA is a matrix protein that plays a crucial role in the c-di-GMP-induced development of robust biofilm and rugose colony in a calcium-dependent manner. Finally, a possible mechanism by which CabA can assemble a functional matrix was also proposed by examining the extracellular complementation of the *cabA* mutant and the *brpA* mutant which lacks EPS, and by determining the influence of calcium on the conformational changes and multimerization of CabA.

## Results

### Identification of the *cabABC* genes

**Fig 1:**
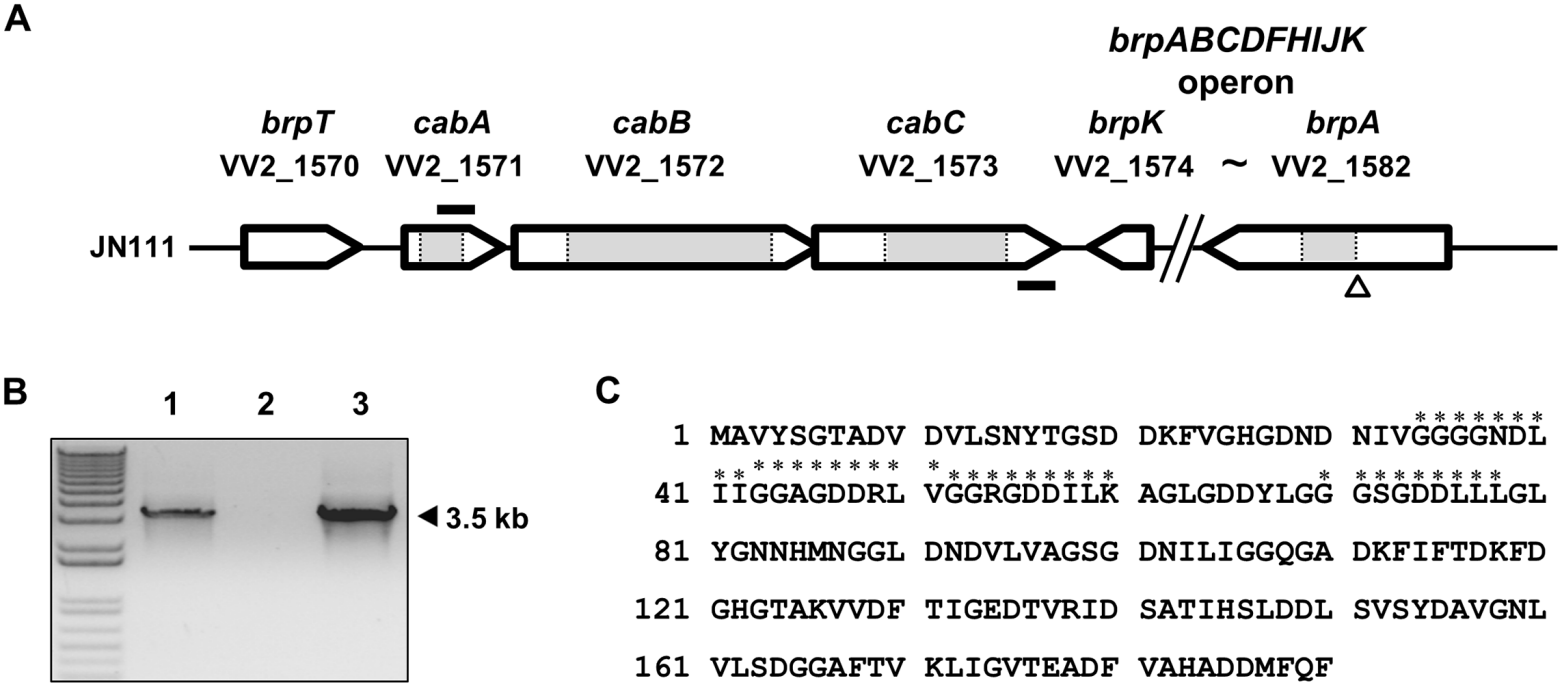
Genetic organization of the *cabABC* operon and amino acid sequence of CabA. (A) The physical map of the *cabABC* operon located between *brpT* and *brpABCDFHIJK* gene cluster. The *open arrows* represent the coding regions and transcriptional directions of the genes. The figure was derived using the *V*. *vulnificus* CMCP6 genome sequences. The gene identifications are shown above each coding region. The size of the *brpABCDFHIJK* operon is reduced as indicated. The *grey boxes* represent the deleted regions of the *cabA*, *cabB*, *cabC*, and *brpA* mutants, respectively. The *nptI* insertion site is indicated as a *triangle*. The primers RTcabA and RTcabC2, used for the RT-PCR analysis, are depicted by *solid bars*. (B) Analysis of the *cabABC* transcript by RT-PCR. Total RNA was isolated from JN111 grown with LBS containing 0.01% arabinose and used for the synthesis of cDNA by reverse transcription. The cDNA (lane 1), DNase I-treated RNA (negative control; lane 2), and genomic DNA (positive control; lane 3) were used as templates for PCR. Molecular size markers (1 kb plus ladder; Invitrogen) and a PCR product are indicated. (C) The amino acid sequence was deduced from the nucleotide sequence of the *cabA* coding region (VV2_1571) and the four putative calcium-binding motifs (GGXG(N/D)DX(L/I/F)X) [28] are indicated with *asterisks*.

A whole-genome microarray analysis was used to compare the transcriptional profiles of the biofilm and planktonic cells of *V*. *vulnificus*. The microarray analysis predicted 321 genes differentially regulated in biofilm cells, of which 177 genes were up-regulated and 144 were down-regulated. A complete list of the 321 genes is shown at Supporting Information (S1 Table). Among the genes that were up-regulated in biofilms, three consecutive genes VV2_1571, VV2_1572, and VV2_1573 were identified and annotated to encode a protein with putative calcium-binding motifs, an ATP-binding cassette (ABC)-type transporter, and a membrane-fusion protein (MFP), respectively (Fig 1A). Since the up-regulation of the three genes in biofilms was not previously reported, their preferential expression was experimentally verified. Quantitative real-time PCR (qRT-PCR) revealed that expression of the three genes increased 2.7–6.8 fold in biofilms (S2 Table). The three genes were named *cabABC* (for calcium-binding protein in biofilms A to C), since the three genes encode proteins involved in the furnishing of a calcium-binding and matrix-associated protein essential for biofilm development as demonstrated in the later parts of this study. It was noteworthy that the *cabABC* genes were 9–69 fold upregulated by elevated intracellular c-di-GMP levels, indicating that *cabABC* genes are regulated by c-di-GMP in cells [25].

### Genetic organization of *cabABC*

Many coding regions that participate in biofilm formation were identified immediately upstream and downstream of the *cabABC* genes from the *V*. *vulnificus* genome sequence (Fig 1A). The *brpABCDFHIJK* operon is located downstream of the *cabC* gene and *brpT* is located upstream of the *cabA* gene. Consequently, the *cabABC* genes are positioned in the intergenic region separating the *brpT* and *brpABCDFHIJK* genes, and are organized in the same orientation as that of *brpT* (Fig 1A). To analyze the expression pattern of the *cabABC* genes at the transcriptional level, cDNA was synthesized from the RNA isolated from JN111 (hereafter referred as a parent strain, constructed to manipulate intracellular c-di-GMP levels as demonstrated in the later parts of this study and S1 Fig) grown with LBS containing 0.01% of arabinose and amplified by PCR using primers RTcabA and RTcabC2 (S3 Table and Fig 1B). As shown in Fig 1B, a single band of, approximately 3.5-kb DNA was amplified. Based on the DNA sequence of *cabABC*, the 3.5-kb PCR product corresponded to the expected size of the amplification product between the RTcabA and RTcabC2 primers. This revealed that *cabA*, *cabB*, and *cabC* are transcribed as a single transcriptional unit. A positive control reaction with *V*. *vulnificus* genomic DNA as a template produced the same-sized 3.5-kb PCR product, and no product was observed when total RNA was directly used as a PCR template (a negative control). Therefore, it appeared that the *cabABC* genes are transcribed as a single operon rather than as three independent genes.

### Sequence analysis of CabA, CabB, and CabC

The amino acid sequence deduced from the *cabA* revealed a protein, CabA, composed of 190 amino acids with a theoretical molecular mass of 19,424 Da and a pI of 3.95 (Fig 1C). CabA revealed a high level of similarity (63% identical in amino acid sequence) with MfpA (http://blast.ncbi.nlm.nih.gov/Blast.cgi), which was previously predicted as a putative calcium-binding protein of *Vibrio parahaemolyticus* [26,27]. The predicted profile of the hydrophobicity (http://web.expasy.org/protscale/) suggested that CabA is a soluble protein rather than a membrane-associated protein. The *in silico* analysis of the amino acid sequences revealed that CabA harbors neither motif nor sequences frequently conserved in the known catalytic enzymes and predicted that CabA is a non-enzymatic structural protein (http://www.ncbi.nlm.nih.gov/cdd). Amino acid sequence analysis of CabA revealed the presence of at least four repeats of the calcium-binding motifs, the acidic glycine-rich nonapeptides, GGXG(N/D)DX(L/I/F)X [28], as depicted in Fig 1C.

The amino acid sequences of *V*. *vulnificus* CabB and CabC were 67 and 65% identical to those of *V*. *parahaemolyticus* MfpB and MfpC, respectively (http://blast.ncbi.nlm.nih.gov/Blast.cgi). MfpB and MfpC have been predicted as an ABC-type transporter and a MFP, respectively, which most likely constitute a type 1 secretion system (T1SS) [26,27]. The predicted profiles of the hydrophobicity (http://web.expasy.org/protscale/) of CabB and CabC indicated that both proteins are membrane-associated proteins. These sequence analyses suggested that CabA is a calcium-binding and non-enzymatic structural protein, and CabB and CabC are membrane-associated proteins homologous to the members of T1SS.

### Calcium binding to CabA

**Fig 2:**
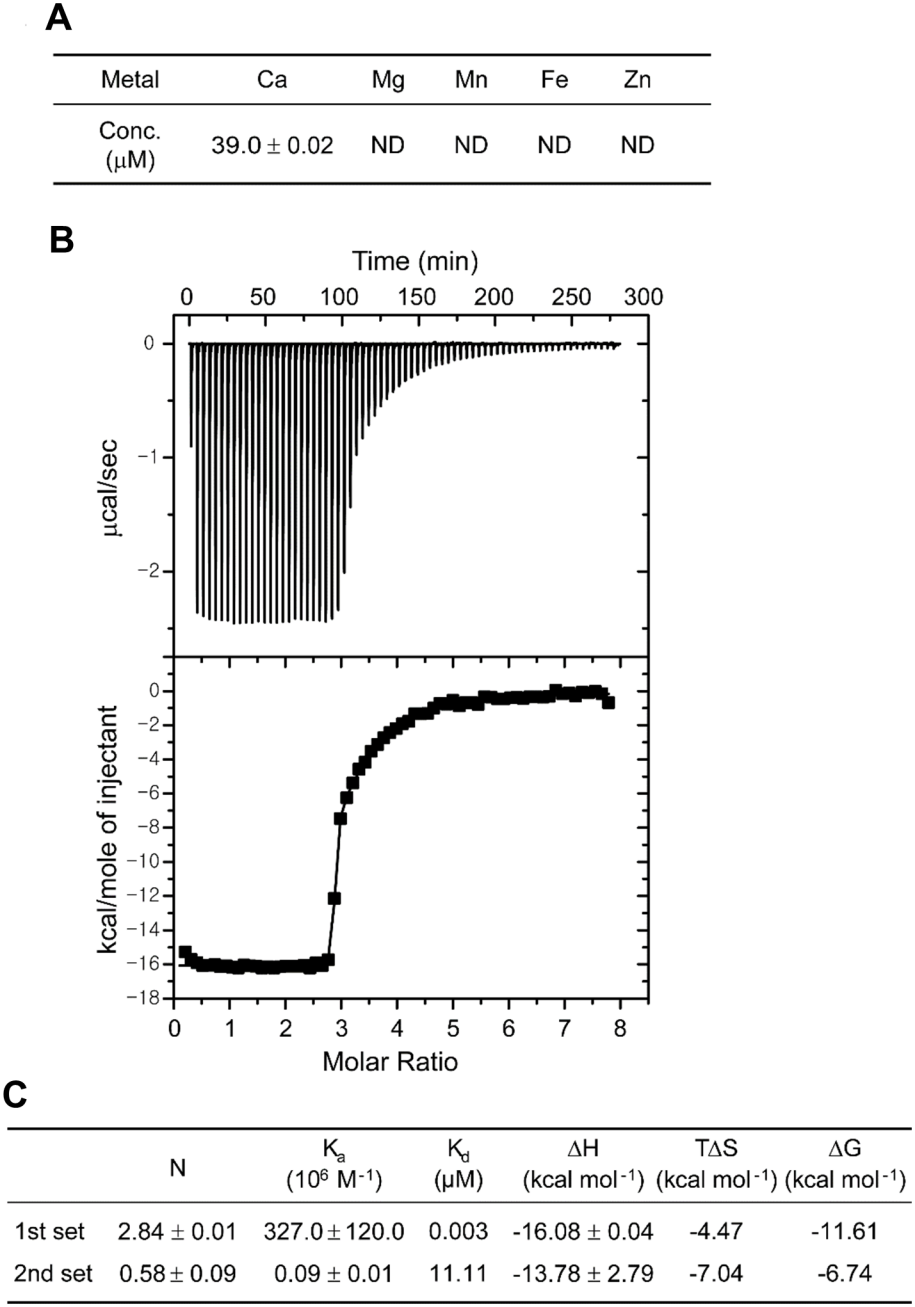
Calcium binding to CabA. (A) The concentrations of metal ions in CabA analyzed by ICP-AES. ND, non-detection. (B) Typical isothermal titration calorimetric measurement of the calcium binding to CabA. The raw data displayed in the upper panel were integrated in the lower panel. The data points represent the experimental injection heat and the solid line corresponds to the calculated fit of the data using a model with two sets of binding sites of the ORIGIN software package (MicroCal Inc.). The heat data of calcium into the reaction buffer was subtracted from the reaction heat data between CabA and calcium. The experimental parameters were as follows: 70 injections of 3 μl of calcium solution, stirring speed of 307 rpm, duration 12 s, and spacing 240 s. (C) Thermodynamic parameters of the calcium binding to CabA.

To examine whether calcium binds specifically to CabA, the metal content of the purified CabA was analyzed using inductively coupled plasma-atomic emission spectrometry (ICP-AES). Only calcium was detected in significant amounts (39 μM) in the protein while other metal elements including magnesium, manganese, iron and zinc were not identified (Fig 2A). The concentration of calcium in the CabA solution (210 μM) indicated that the molar ratio of calcium ion bound to CabA was about 0.19. However, it is noteworthy that the recombinant CabA protein was synthesized in the *Escherichia coli* cytosol which contains limited concentration of calcium ion around 90 nM [29]. Thus, the molar ratio of calcium to CabA determined by the ICP-AES does not necessarily reflect actual stoichiometry of calcium bound to CabA. To evaluate calcium-binding parameters for CabA, isothermal titration calorimetry (ITC) experiments were carried out. The calcium-free CabA protein was titrated with calcium, and the ITC data were integrated and fit into a binding model consisting of two sets of binding sites which yields three very tight (*K*_d_ = 3 nM) and one weaker (*K*_d_ = 11 μM) sites (Fig 2B). The thermodynamic parameters of calcium binding to CabA determined by ITC are summarized in Fig 2C.

### Construction of JN111 and effects of c-di-GMP on the expression of *cabA*

**Fig 3:**
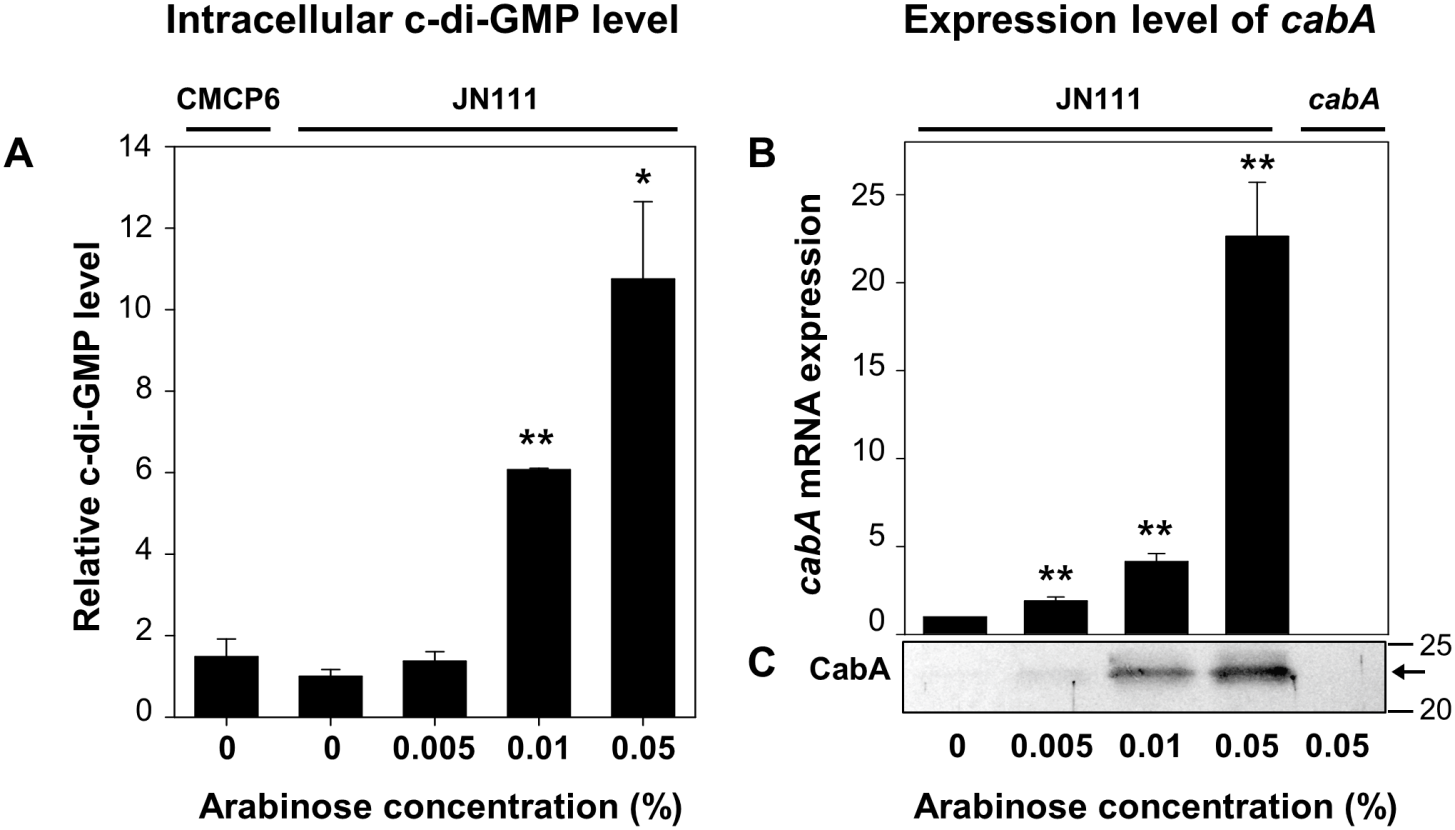
Intracellular levels of c-di-GMP and *cabA* expression. The strains were grown to *A*_*600*_ of 0.15 with VFMG-CF containing 10 mM of CaCl_2,_ and different concentrations of arabinose as indicated. (A) Intracellular levels of c-di-GMP were measured using LC-MS. (B) Transcripts of *cabA* were determined using qRT-PCR. Relative levels of the c-di-GMP and *cabA* mRNA were presented as those levels in JN111 grown without arabinose as 1, respectively. *, *P*<0.05; **, *P*<0.005 relative to the c-di-GMP or *cabA* mRNA levels in JN111 grown without arabinose. Error bars represent the SD. (C) Protein samples were resolved on SDS-PAGE and immunoblotted using the rabbit anti-CabA antibody. The protein size markers (Precision Plus Protein Standards; Bio-Rad Laboratories) and CabA (*arrows*) are shown in kDa. CMCP6, wild type; JN111, parent strain; *cabA*, *cabA* mutant.

Since expression of the *cabABC* genes is regulated by c-di-GMP [25], *V*. *vulnificus* JN111, in which *dcpA* encoding a DGC is controlled under P_*BAD*_, was constructed to manipulate the intracellular c-di-GMP levels (Materials and Methods and S1 Fig). The intracellular c-di-GMP levels of JN111, determined using liquid chromatography–mass spectrometry (LC-MS) [30], were gradually elevated as concentrations of arabinose increased in the growth media (Fig 3A). The intracellular c-di-GMP level of the wild type CMCP6, in which the expression of *dcpA* was not manipulated, was approximately equivalent to the uninduced (or induced with 0.005% arabinose) JN111 level, indicating that the over-expression of *dcpA* leads to the increase of c-di-GMP well above the wild type level (Fig 3A). The results indicated that JN111 was an appropriate strain to manipulate intracellular c-di-GMP levels. The cellular levels of the *cabA* transcript and CabA protein in JN111 grown with VFMG-CF (the modified *V*. *fischeri* minimal medium as described in Materials and Methods) containing different concentrations of arabinose were also determined. In the absence of arabinose, the qRT-PCR and Western blot analyses revealed little, if any, expression of *cabA*. However, *cabA* expression increased along with the increased concentrations of arabinose in the growth media (Fig 3B and 3C). The combined results confirmed that *cabA* expression is induced by c-di-GMP and the increased expression of *cabA* in biofilms, as observed in microarray analysis, was probably attributed to elevated c-di-GMP. Strikingly, the presence of more than 0.02% arabinose was detrimental to the biofilm formation of the *V*. *vulnificus* strains (S2 Fig). Therefore, 0.01 and 0.02% arabinose were used in the hereafter experiments for biofilm and colony morphology analyses, respectively.

### Effects of the *cabA* mutation on biofilm formation

**Fig 4:**
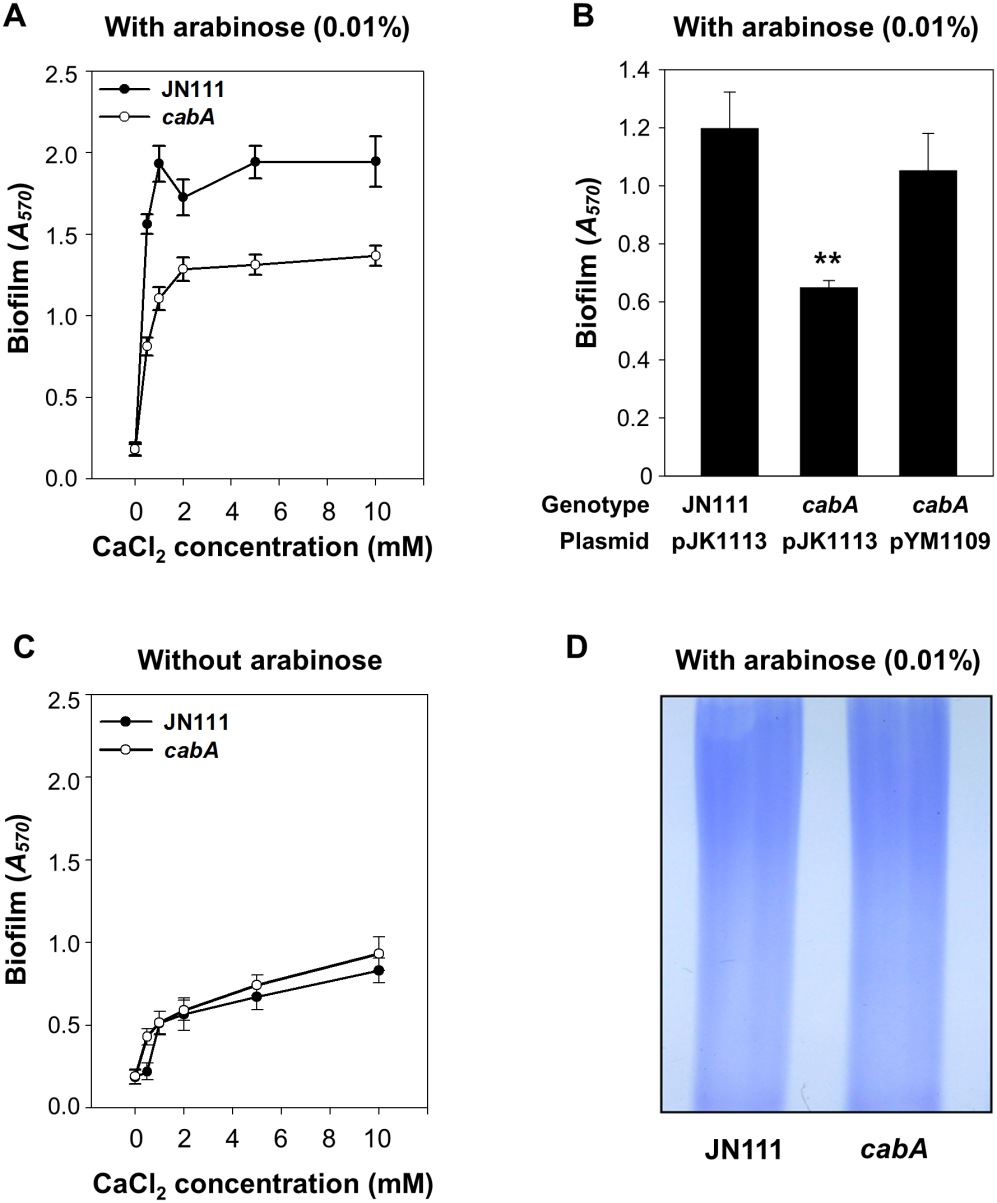
Effects of the *cabA* mutation on biofilm formation. (A and C) Biofilms of the strains were grown for 24 h on 96-well microtiter plate wells containing VFMG-CF supplemented with or without 0.01% arabinose and with various levels of CaCl_2_, and quantified using CV staining. (B) Biofilms of the strains were grown with VFMG-CF containing 0.01% arabinose and 10 mM CaCl_2_, and quantified as described above. Ampicillin and kanamycin (100 μg/ml for each) were used to maintain plasmids in the strains. JN111 (pJK1113), parent strain; *cabA* (pJK1113), *cabA* mutant; *cabA* (pYM1109), complemented strain. **, *P*<0.005 relative to the JN111 biofilm. Error bars represent the SD. (D) EPS extracts were prepared from the strains grown on the LBS agar plates supplemented with 0.02% arabinose and resolved on a 4% stacking SDS-PAGE. JN111, parent strain; *cabA*, *cabA* mutant.

Biofilms of JN111 and the isogenic *cabA* mutant were formed in microtiter plates, and quantified using crystal violet (CV) staining assays (Fig 4A,4B and 4C). CV staining revealed that the level of biofilm formation is consistently reduced in the *cabA* mutant under conditions of elevated c-di-GMP (0.01% arabinose) (Fig 4A). This difference in the biofilm-forming ability of JN111 and the *cabA* mutant was apparent when increasing concentrations of calcium were present in the growth media (Fig 4A). These results indicated that CabA induced by c-di-GMP plays a role in biofilm formation in a calcium-dependent manner. Complementation with a functional *cabA* gene (pYM1109) restored the biofilm-forming ability of the *cabA* mutant to the level comparable to that of JN111 (Fig 4B). Therefore, the reduced biofilm-forming ability of the *cabA* mutant resulted from the inactivation of functional *cabA* rather than any polar effects on genes downstream of *cabA*. Noticeably, when grown in the absence of arabinose, JN111 as well as the *cabA* mutant produced very low levels of biofilm (Fig 4C). Furthermore, no obvious differences in the amounts of biofilm formed by JN111 and the *cabA* mutant in the absence of arabinose were observed. This indicated that the trace amounts of CabA expressed in the cells grown in the absence of arabinose would be not sufficient to contribute to biofilm formation (Fig 3B). Biofilm-forming abilities of the strains, in the same conditions used for Fig 4A and 4C but in a larger scale in test tubes, were similar to those observed in microtiter plates as determined by the pictures of the resulting CV staining (S3 Fig). These results demonstrated that CabA contributes to the c-di-GMP-mediated biofilm formation and requires calcium for its optimum activity.

The decreased formation of biofilm by the *cabA* mutant could be indicative of defects in the production of EPS which is known to be a major component of the *Vibrio* spp. biofilm matrix [4]. However, the amount of total EPS isolated from JN111 was indistinguishable from that isolated from the *cabA* mutant when determined based on the intensities of EPS bands resolved on the SDS-PAGE gel (Fig 4D). The result implied that CabA did not significantly influence the levels of EPS produced.

### CabB- and CabC-dependent secretion of CabA into extracellular matrix

**Fig 5:**
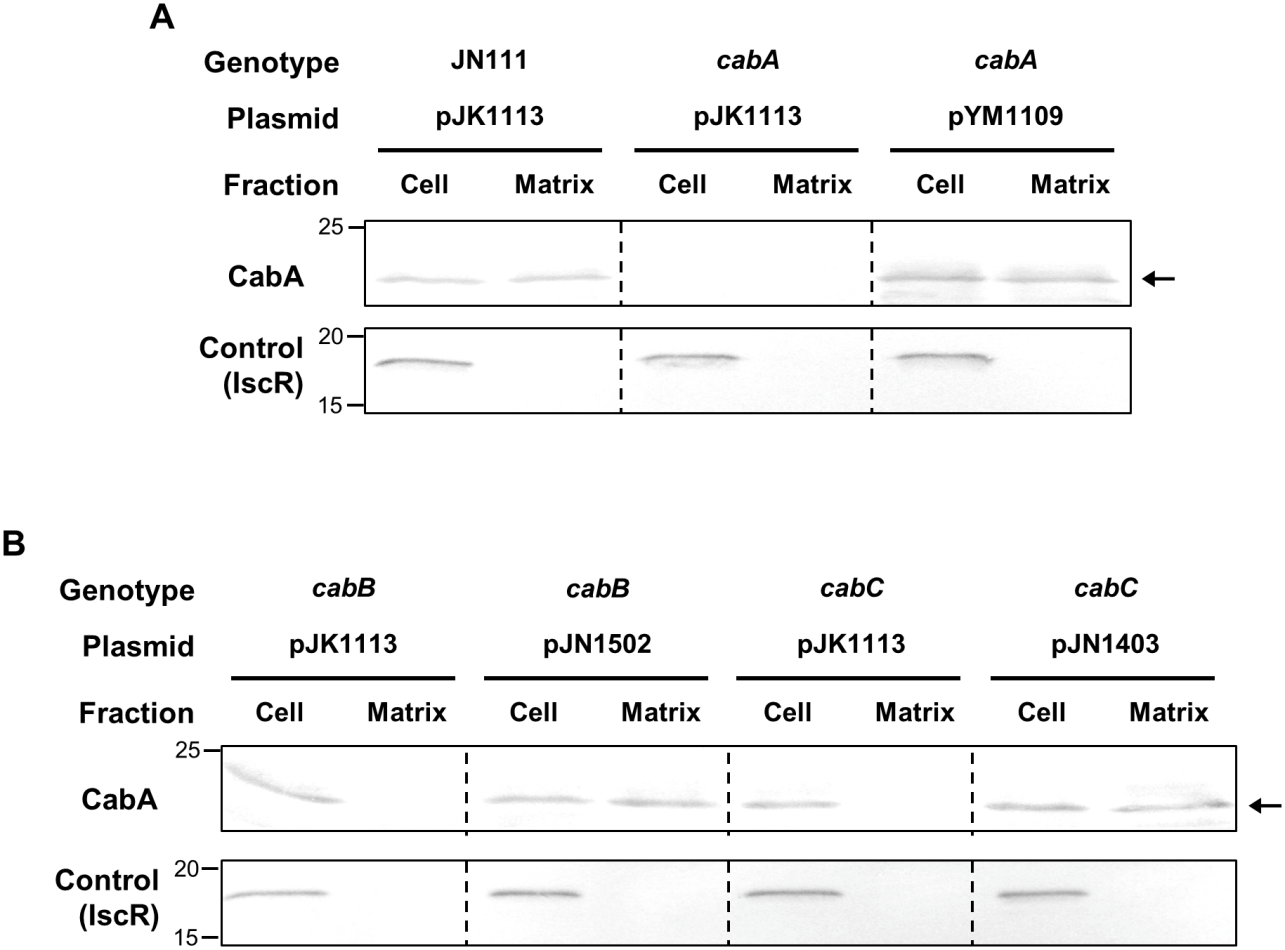
CabA in the cell and matrix fraction of biofilms. Biofilms of the strains were grown in the flask containing VFMG-CF supplemented with 0.01% of arabinose and 10 mM CaCl_2_. (A and B, upper panels) Cell and matrix fractions of the biofilms were prepared and the resulting fractions, equivalent to 10 μg of total proteins, were resolved on SDS-PAGE and immunoblotted using the rabbit anti-CabA antibody. (A and B, lower panels) In order to demonstrate that cells were not lysed during the fractionation procedures, an intracellular regulator IscR was detected in each fraction using the rabbit anti-IscR antibody prepared previously [31]. The protein size markers (Precision Plus Protein Standards; Bio-Rad Laboratories) and CabA (*arrows*) are shown in kDa. JN111 (pJK1113), parent strain; *cabA* (pJK1113), *cabA* mutant; *cabA* (pYM1109), complemented strain; *cabB* (pJK1113), *cabB* mutant; *cabB* (pJN1502), complemented strain; *cabC* (pJK1113), *cabC* mutant; *cabC* (pJN1403), complemented strain; Cell, cell fraction; Matrix, matrix fraction.

To get a better understanding on the role of CabA in biofilm formation, the localization of the protein in the biofilms was determined. Biofilms were fractionated to separate cells from the extracellular matrix and subjected to Western blot analyses. As evident from the result shown in Fig 5A, CabA was detected in the matrix fraction as well as the cell fraction of the JN111 biofilms, indicating that CabA is a protein secreted to the cell exterior in the matrix of biofilms. In contrast, CabA was detected neither in the cell nor the matrix fractions of the *cabA* mutant biofilms. This lack of CabA in the *cabA* mutant biofilms was restored by the introduction of pYM1109. To verify that the fractionation was performed without lysis of cells, a cytosolic regulatory protein IscR [31] was determined in each fraction by Western blot analysis. The result that IscR was found exclusively in the cell fraction ruled out the possibility that CabA in the matrix fraction resulted from cell lysis (Fig 5A).

As mentioned above, the deduced amino acid sequences of CabB and CabC share homology with those of MfpB and MfpC which are predicted to constitute a T1SS [26,27]. This information prompted us to examine whether CabA secretion to the cell exterior indeed depends on the presence of functional CabB and CabC. For this purpose, the isogenic *cabB* and *cabC* mutants were constructed (Table 1) and the CabA localization in biofilms was determined (Fig 5B). Western blot analyses revealed that CabA was detected exclusively in the cell fraction, but not in the matrix fraction, of the *cabB* and *cabC* mutant biofilms. The failure to detect CabA in the matrix fraction is indicative of defects in the secretion of CabA. The defect of CabA secretion in the *cabB* and *cabC* mutant was restored by the introduction of pJN1502 or pJN1403 carrying either recombinant *cabB* or *cabC*, respectively. IscR was detected once again exclusively in the cell fraction (Fig 5B). The results indicated that CabB and CabC play a critical role in the secretion of CabA, possibly by composing a T1SS. Importantly, the *cabB* and *cabC* mutant failed to accumulate additional CabA in the cell fraction as determined based on band intensity (Fig 5B). This was not surprising since previous studies have shown that the T1SS-impaired strains prevent accumulation of the protein substrates (effectors) in cells by degrading them by an unknown mechanism [32,33]. This observation was not pursued further. These combined results suggested that CabA is a secreted protein and localized at the extracellular matrix.

**Table 1:** Bacterial strains and plasmids used in this study.

Strain or plasmid	Relevant characteristics^a^	Reference or source
**Bacterial strains**		
*V*. *vulnificus*		
CMCP6	Wild type *V*. *vulnificus*, virulent	Laboratory collection
YM112	CMCP6 with Δ*cabA*	This study
JN111	CMCP6 with P_*BAD*_-*dcpA*	This study
YM112D	JN111 with Δ*cabA*	This study
JN151D	JN111 with Δ*cabB*	This study
JN141D	JN111 with Δ*cabC*	This study
JN094D	JN111 with Δ*brpA*::*nptI*; Km^r^	This study
JN143D	JN111 with Δ*cabA*, Δ*brpA*::*nptI*; Km^r^	This study
*E*. *coli*		
BL21 (DE3)	F^-^ *ompT hsdS*_*B*_ (r_B_^-^, m_B_^-^) *gal dcm* (DE3)	Laboratory collection
DH5α	*supE44* Δ*lacU169 (*ϕ80*lacZ* ΔM15*) hsdR17 recA1 endA1 gyrA96 thi-1 relA1*; plasmid replication	Laboratory collection
S17-1λ*pir*	λ-*pir* lysogen; *thi pro hsdR hsdM* ^+^ *recA* RP4-2 Tc::Mu-Km::Tn7;Tp^r^ Sm^r^; host for π-requiring plasmids; conjugal donor	[63]
**Plasmids**		
pDrive	PCR product cloning vector; Ap^r^ Km^r^	Qiagen
pDM4	Suicide vector; *ori* R6K; Cm^r^	[56]
pBAD24	Expression vector with the P_*BAD*_ promoter; Ap^r^	[49]
pJN1105	pDrive with P_*dcpA*_-*dcpA*; Ap^r^ Km^r^	This study
pJN1106	pDrive with P_*dcpA*_-P_*BAD*_-*dcpA*; Ap^r^ Km^r^	This study
pJN1107	pDM4 with P_*dcpA*_-P_*BAD*_-*dcpA*; Cm^r^	This study
pET28a(+)	His-tag protein expression vector; Km^r^	Novagen
pYM1202	pET28a(+) with *cabA*; Km^r^	This study
pYM1102	pDM4 with Δ*cabA*; Cm^r^	This study
pJN1504	pDM4 with Δ*cabB*; Cm^r^	This study
pJN1402	pDM4 with Δ*cabC*; Cm^r^	This study
pJN0907	pDM4 with Δ*brpA*::*nptI*; Cm^r^, Km^r^	This study
pJK1113	pKS1101 with *nptI*; Ap^r^ Km^r^	[57]
pYM1109	pJK1113 with *cabA*; Ap^r^ Km^r^	This study
pJN1502	pJK1113 with *cabB*; Ap^r^ Km^r^	This study
pJN1403	pJK1113 with *cabC*; Ap^r^ Km^r^	This study

^a^ Km^r^, kanamycin resistant; Tp^r^, trimethoprim resistant; Sm^r^, streptomycin resistant; Cm^r^, chloramphenicol resistant; Ap^r^, ampicillin resistant.

### *In situ* determination of the CabA localization in biofilms

**Fig 6:**
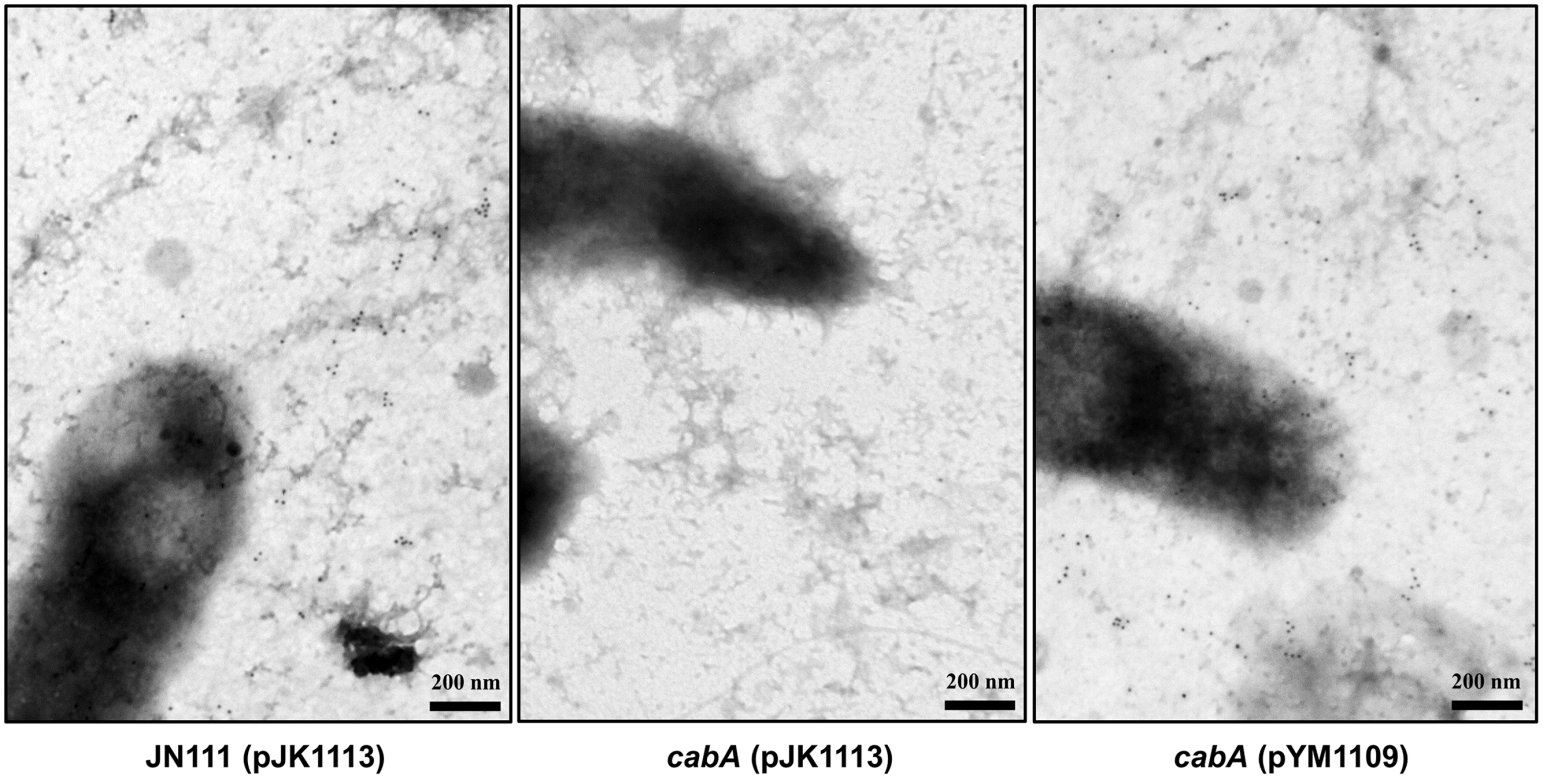
Distribution of CabA in biofilms. Biofilms of the strains were grown on the nickel grids containing VFMG-CF supplemented with 0.01% of arabinose and 10 mM of CaCl_2_. CabA in the biofilms was detected with the rabbit anti-CabA antibody, labeled with the secondary antibody conjugated to 10-nm gold particles, and then visualized using TEM (JEM1010, JEOL) at a 40000× magnification. Black dots represent the CabA protein labeled with the antibody conjugated to gold particles. Bars, 200 nm. JN111 (pJK1113), parent strain; *cabA* (pJK1113), *cabA* mutant; *cabA* (pYM1109), complemented strain.

The proteins secreted to the cell exterior might perform diverse functions such as initial attachment to surfaces and subsequent formation of matrix. It has been reported that proper localization of the proteins is crucial for their functions in biofilm formation [34]. Many, if not all, proteins involved in the initial surface attachment, namely adhesins, are somehow retained on the cell envelope as observed with *Pseudomonas fluorescens* LapA and *Salmonella enteritidis* SiiE [35,36]. Therefore, to examine whether CabA is another adhesin, biofilms of the strains were formed on nickel grids and the localization of the functional form of CabA was determined *in situ* with a rabbit anti-CabA antibody and labeled with gold particles. When visualized using transmission electron microscopy (TEM), secreted CabA proteins were not concentrated or localized along the cell boundaries, but rather distributed distally from the cells throughout the extracellular medium of the biofilm matrix (Fig 6). In contrast, CabA was not detected at all in the *cabA* mutant biofilm, and this absence of CabA was restored when the *cabA* mutant was complemented with pYM1109. The throughout, albeit not uniform, distribution of CabA in the extracellular matrix indicated that CabA is not a cell envelope-localized adhesin. We therefore hypothesize that CabA is a structural component of the extracellular matrix.

### Kinetics of biofilm formation and *cabA* expression

**Fig 7:**
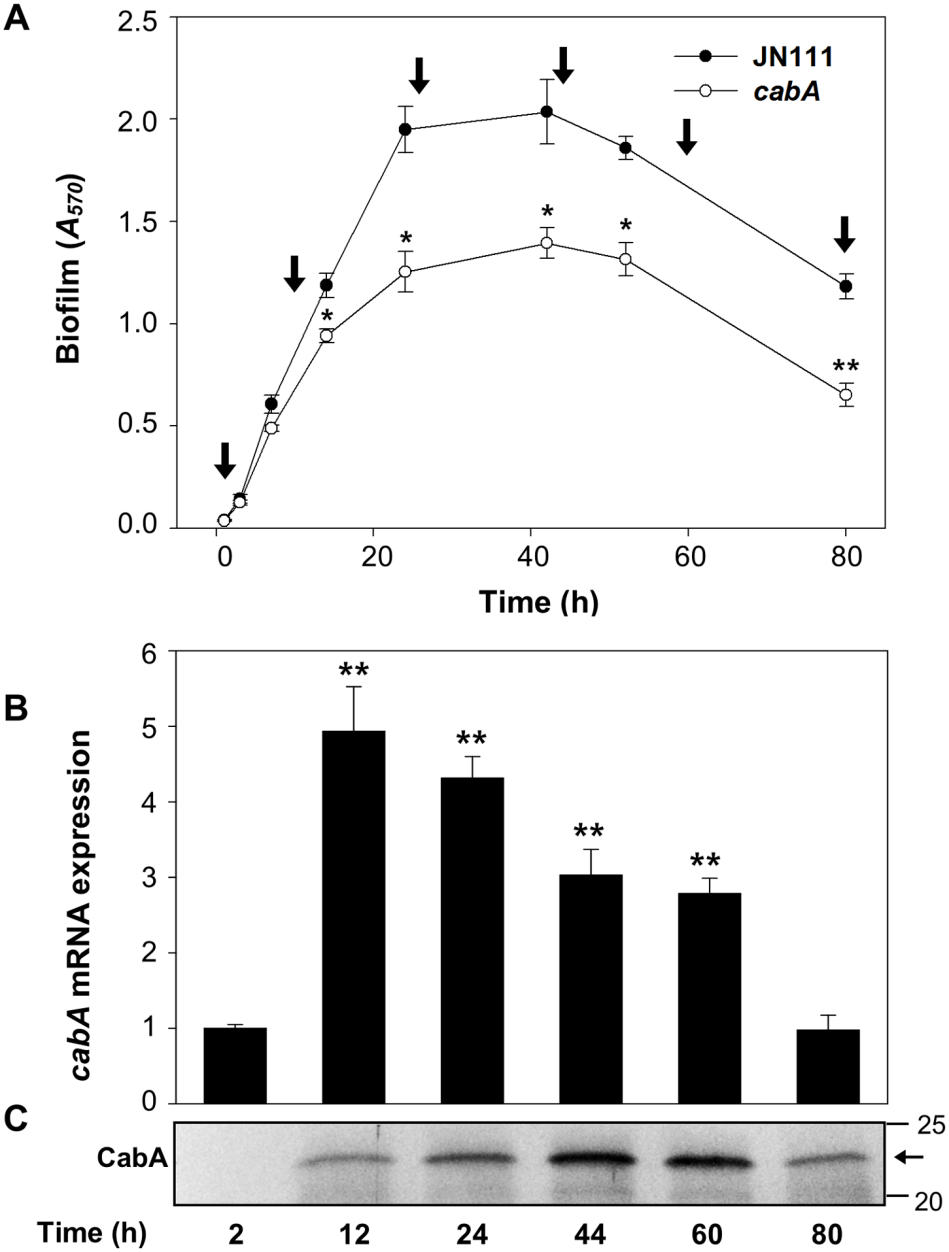
Expression of CabA at the different development stages of biofilm. (A) Biofilm development of JN111 and the *cabA* mutant with VFMG-CF containing 10 mM of CaCl_2_ and 0.01% of arabinose was monitored using CV staining. Total RNAs and proteins were prepared from the JN111 biofilms (that is, from the cells and matrices) harvested at different stages as indicated by *vertical arrows*. *, *P*<0.05; **, *P*<0.005 relative to the JN111 biofilm. JN111, parent strain; *cabA*, *cabA* mutant. (B) The *cabA* mRNA levels were determined by qRT-PCR analyses, and the *cabA* mRNA level in the biofilms harvested after 2 h incubation was set as 1. **, *P*<0.005 relative to the *cabA* mRNA level in the biofilms harvested after 2 h incubation. Error bars represent the SD. (C) Protein samples were resolved on SDS-PAGE and immunoblotted using the rabbit anti-CabA antibody. The protein size markers (Precision Plus Protein Standards; Bio-Rad Laboratories) and CabA (*horizontal arrow*) are shown in kDa.

Biofilm development of JN111 and *cabA* mutant was assessed at different time points using CV staining. Biofilm formation of both JN111 and the *cabA* mutant initiated at 2 h, reached maximum levels at 44 h, and then steadily decreased during extended periods of incubation (Fig 7A). During the first 10 h of biofilm development, the amounts of both biofilms of JN111 and the *cabA* mutant were not significantly different. In contrast, the biofilm formation of the *cabA* mutant was consistently reduced than that of JN111 after 10 h. The results indicated that CabA is not crucial for the initial surface attachment but plays an important role in the subsequent growth and maturation of biofilms. Consistent with this, *cabA* expression increased greatly at both transcript and protein levels after 12 h (Fig 7B). Interestingly, the CabA protein increased up to 44 h whereas the *cabA* transcript arrived at the maximum at 12 h and then decreased (Fig 7C). The prolonged increase of CabA may result from the accumulation of the protein in the extracellular matrix during biofilm development as observed in *Pseudomonas putida* biofilms [37]. These results indicated that CabA is a matrix-associated protein and plays a crucial role as the biofilm matures.

### Effects of the *cabA* mutation on colony morphology

**Fig 8:**
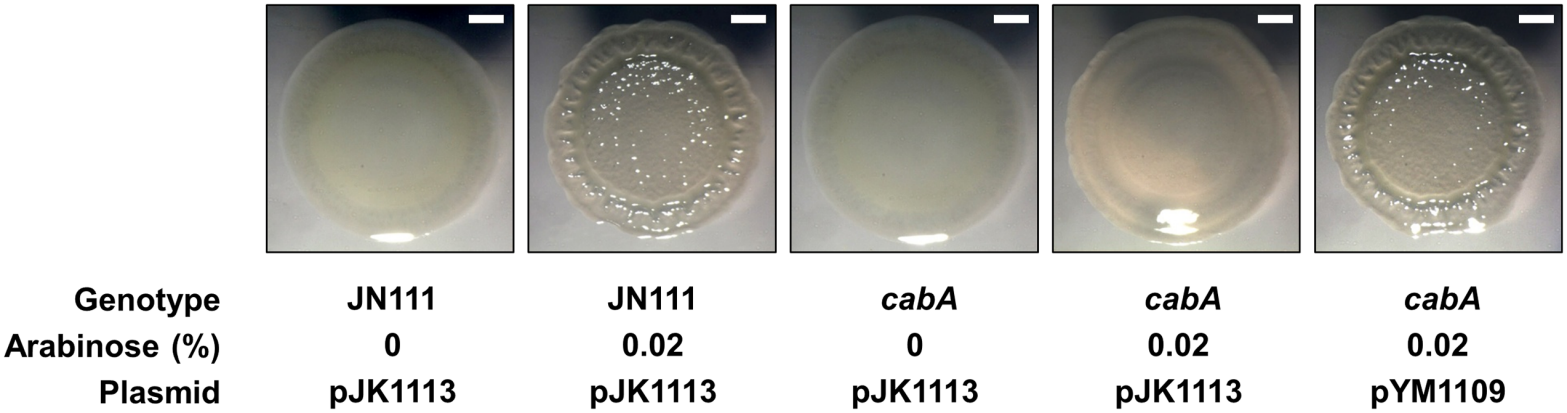
Effects of the *cabA* mutation on colony morphology. (A) The strains were spotted onto VFMG-CF agar plates supplemented with or without 0.02% of arabinose and 10 mM of CaCl_2_. Then, the plates were incubated at 30°C for 3 d. Ampicillin and kanamycin (200 μg/ml for each) were used to maintain plasmids in the strains. Each colony representing the mean rugosity of colony morphology from at least three independent experiments was visualized using a stereomicroscope (Stemi DV4, Zeiss) at an 8× magnification. Bars, 1 mm; JN111 (pJK1113), parent strain; *cabA* (pJK1113), *cabA* mutant; *cabA* (pYM1109), complemented strain.

In order to determine the role of CabA on colony morphology, JN111 and the *cabA* mutant were grown on the VFMG-CF agar plate supplemented with different concentrations of arabinose and 10 mM of CaCl_2_ (Fig 8). When grown in the absence of arabinose, JN111 revealed smooth colony morphology. However, the smooth colony morphology of JN111 switched to rugose (wrinkled) in the presence of 0.02% of arabinose, indicating that elevating c-di-GMP induced the development of the rugose colony morphology. In contrast to JN111, the *cabA* mutant formed smooth colony morphology regardless of the presence of arabinose and the smooth colony morphology of the *cabA* mutant did not switch to rugose under conditions of elevated c-di-GMP (Fig 8). These results indicated that the rugose colony formation induced by the elevated c-di-GMP is CabA-dependent. Complementation of the *cabA* mutant by the introduction of pYM1109 carrying a recombinant *cabA* shifted the smooth colony morphology of the *cabA* mutant to the rugose comparable to that of JN111 (Fig 8). Since rugose colony morphology frequently reflects increased levels of EPM [4], these results reinforce the view that CabA is an important constituent of the *V*. *vulnificus* biofilm matrix.

### Effects of the *cabA* mutation on biofilm structure

**Fig 9:**
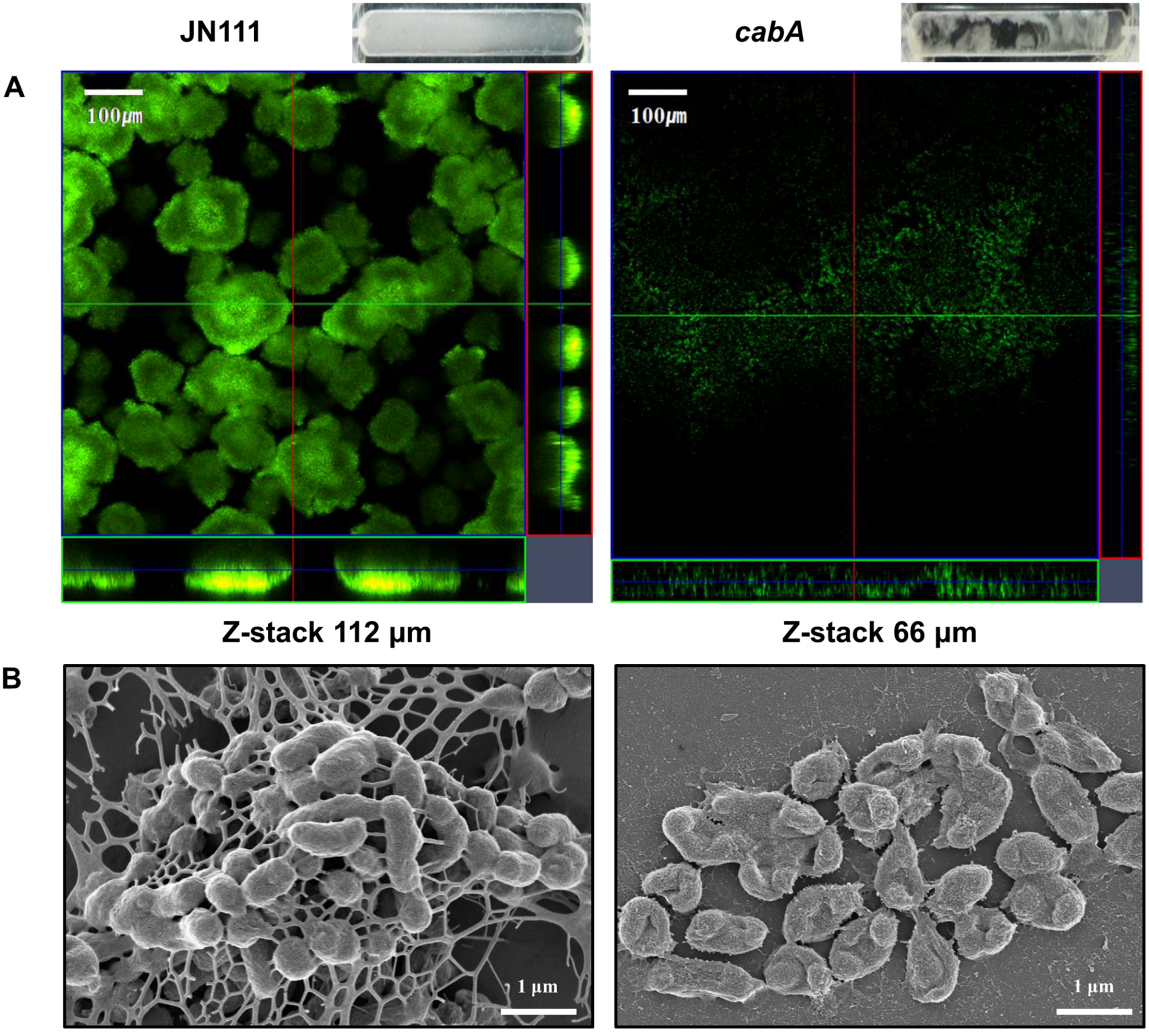
Effects of the *cabA* mutation on biofilm structure. Biofilms of the strains were grown for 3 d in the flow cells containing VFMG-CF supplemented with 0.01% arabinose and 10 mM CaCl_2_. (A) Biofilms were stained by LIVE/DEAD *Bac*Light Viability Kit (Invitrogen), and CSLM (LSM710, Zeiss) images were acquired at a 100× magnification. The depth of the Z-stack is indicated below the images in μm. Images of flow cell chambers were presented on the upper-right corner of each CSLM image. (B) Biofilms of the strains were fixed, dehydrated, coated with platinum, and visualized using SEM (Supra 55VP, Zeiss) at a 30000× magnification. Bars, 100 μm (A) and 1 μm (B); JN111, parent strain; *cabA*, *cabA* mutant.

The biofilm biomass of JN111 was less than 2-fold greater than that of the *cabA* mutant as determined by CV staining (Fig 4A and 4B). This limited CabA-dependent increase of biofilm formation led us to examine whether the *cabA* mutation has effects on the structure of biofilm. The biofilms produced on the coverslips in flow cells were stained using LIVE/DEAD *Bac*Light Viability Kit (Invitrogen, Carlsbad, CA) and visualized by confocal scanning laser microscopy (CSLM). As shown in Fig 9A, JN111 displayed the structure of robust, large, uniform, and mushroom-shaped biofilms, whereas the *cabA* mutant produced weak, sparse, inconsistent, and markedly unstructured biofilms. JN111 formed much thicker biofilms than the *cabA* mutant in flow cells, and Z-stack measurements indicated that the depths of the JN111 and the *cabA* mutant biofilms were about 112 and 66 μm, respectively. When determined by the flow cell images, the JN111 biofilms were stable and evenly covered the glass coverslip, whereas the *cabA* mutant biofilms seemed to be easily washed away and disintegrated by medium flow, which resulted in uncovered surfaces of the glass coverslip (Fig 9A, upper-right corner). The results indicated that the structure of the CabA-deficient matrix is not robust enough to withstand medium flow in flow cells, and CabA is essential more for the structure rather than the quantity of the *V*. *vulnificus* biofilm.

To further investigate the role of CabA in biofilm formation, biofilm structures of the strains were examined in more detail using scanning electron microscopy (SEM) (Fig 9B). JN111 produced compact and thick biofilms in which bacterial cells were bound together and encased within extracellular matrices. Filamentous materials that connect the bacterial cells to each other were visible within the extracellular matrix of JN111. In contrast, the *cabA* mutant cells were scattered without any extracellular filaments binding the cells together. The *cabA* mutant formed only few bacterial cell clusters that were left nearly bare, albeit the occasional appearance of thread-like strands. The observation that the *cabA* mutant made no matrices at all emphasizes the importance of CabA to the extracellular matrix formation. Since the amounts of EPS produced by JN111 and the *cabA* mutant grown on the LBS agar plates were not significantly different as described above (Fig 4D), it is reasonable to conclude that CabA appears to be important specifically for the structure of the extracellular matrix.

**Fig 10:**
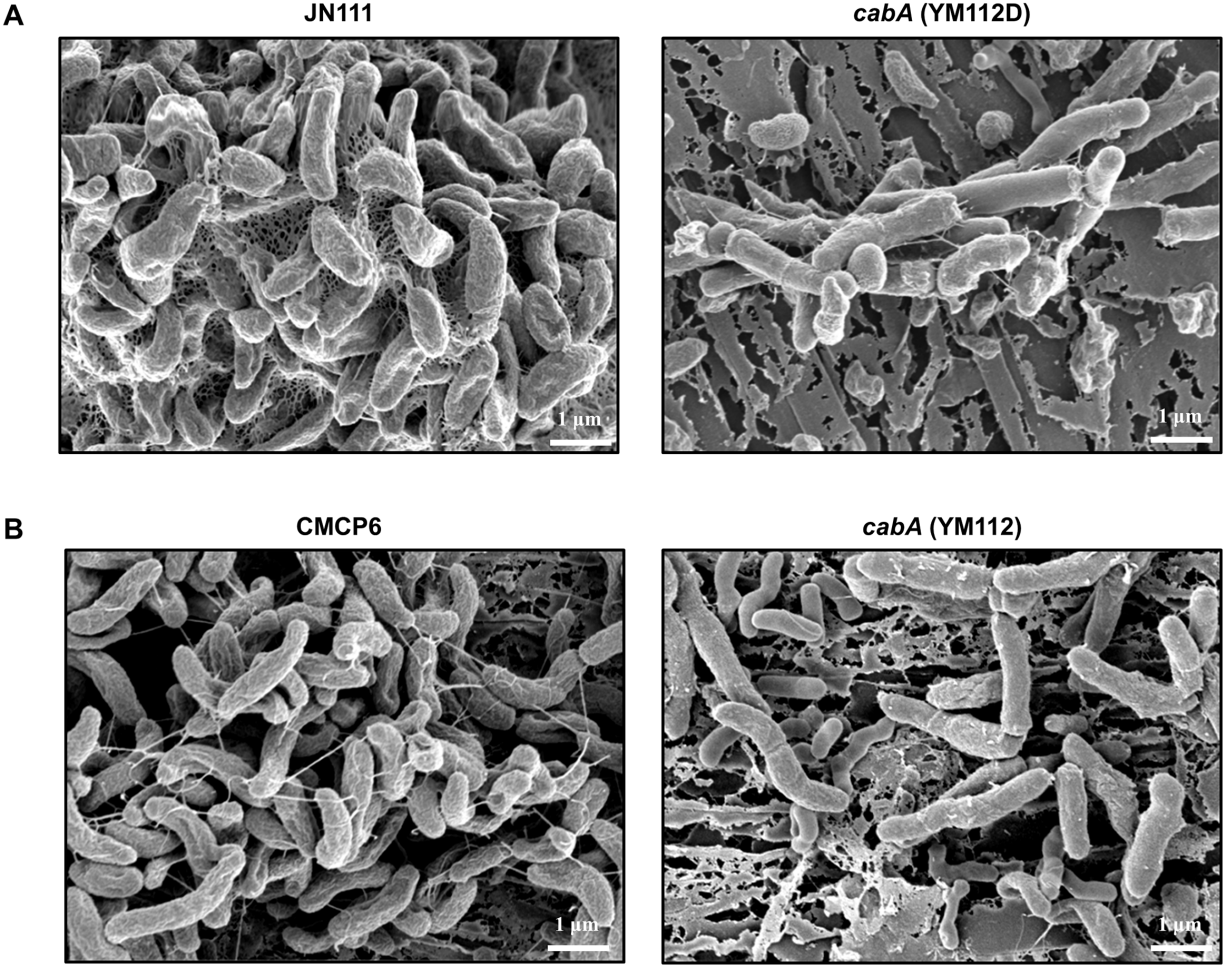
Structure of biofilms formed on oyster shells. Biofilms of JN111 and its isogenic mutant (A) or CMCP6 and its isogenic mutant (B) were grown on oyster shells for 24 h in 24-well microtiter plates containing VFMG-CF supplemented with or without 0.01% arabinose and with 10 mM CaCl_2_. The biofilms were fixed, dehydrated, coated with platinum, and visualized using SEM (Supra 55VP, Zeiss) at a 20000× magnification. Bars, 1 μm; JN111, parent strain; YM112D, *cabA* mutant (of JN111); CMCP6, wild type; YM112, *cabA* mutant (of CMCP6).

Biofilms of the strains were grown on oyster shells and their structures were examined using SEM (Fig 10). As observed in flow cells, JN111 formed robust biofilms on the oyster shells with filamentous materials connecting bacterial cells in the extracellular matrix whereas its isogenic *cabA* mutant (YM112D) formed only few bacterial cell clusters with the significantly smaller amount of extracellular filaments (Fig 10A). Similarly, the wild type CMCP6, in which the intracellular c-di-GMP levels were not manipulated, produced markedly greater amounts of extracellular matrix compared to its isogenic *cabA* mutant (YM112) (Fig 10B). Both *cabA* mutants, YM112D and YM112, were not able to develop considerable amounts of multicellular layers of bacterial cells on the surfaces of oyster shells. These results, combined with the results of Fig 9A and 9B, suggested that CabA is essential for the structural integrity of the *V*. *vulnificus* biofilms in the natural environments as well as *in vitro*.

### Exogenous addition of purified CabA rescues the *cabA* mutant

**Fig 11:**
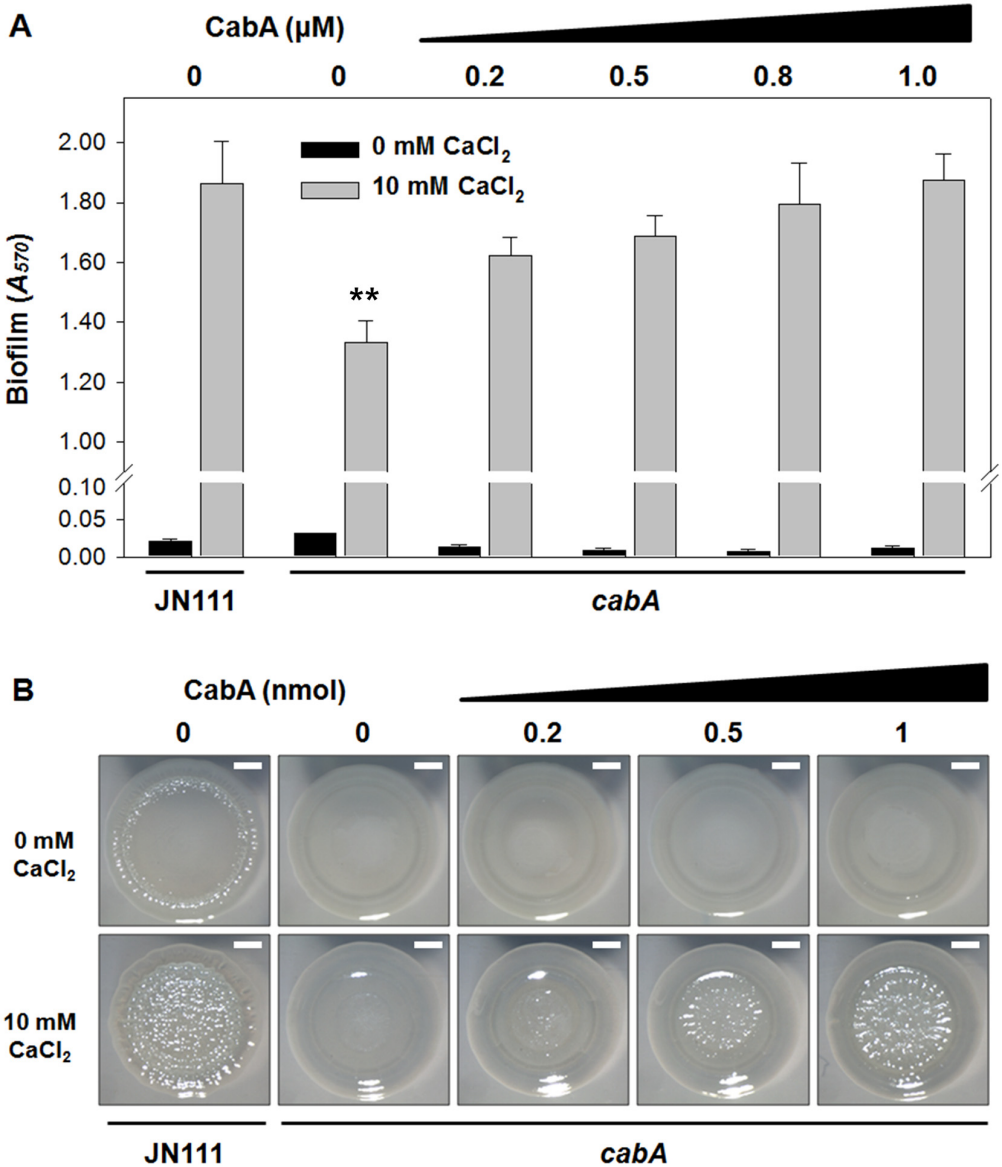
Effects of purified CabA protein on biofilm formation and colony morphology. (A) Biofilms of the strains were grown with VFMG-CF supplemented with 0.01% arabinose, and various amounts of exogenously-provided calcium-free CabA in the absence or presence of 10 mM CaCl_2_. After 24 h incubation, biofilms were quantified using CV staining. Error bars represent the SD. **, *P*<0.005 relative to the JN111 biofilm grown with 10 mM CaCl_2_. (B) The strains were spotted onto VFMG-CF agar plates supplemented with 0.02% of arabinose and with or without 10 mM CaCl_2_. After being grown for 12 h at 30°C, calcium-free CabA were added exogenously onto the growing colonies as indicated and then further incubated for 2.5 d. Each colony representing the mean rugosity of colony morphology from at least three independent experiments was visualized using a stereomicroscope (Stemi DV4, Zeiss) at an 8× magnification. Bars, 1 mm; JN111, parent strain; *cabA*, *cabA* mutant.

We examined if exogenous CabA can rescue the biofilm defect of the *cabA* mutant. The *cabA* mutant in microtiter plates was allowed to form static biofilm in the presence of increasing amounts of purified CabA. Purified CabA was able to restore the biofilm- and rugose colony forming ability of the *cabA* mutant (Fig 11). The *cabA* mutant incubated in the presence of purified CabA of more than 0.8 μM formed biofilms that were comparable to that of JN111, in terms of their biomass (Fig 11A). This CabA complementation of the *cabA* mutant to form biofilm exhibited dependence on calcium and was not apparent unless calcium was present in the growth media (Fig 11A). Similarly, the smooth colony morphology of the *cabA* mutant on VFMG-CF agar plate was shifted to rugose in the presence of increasing amounts of CabA (Fig 11B). However, this CabA complementation of the *cabA* mutant to develop the rugose colony morphology was not obtainable when calcium was not added to the growth media (Fig 11B). The rugose colony morphology developed by JN111 exhibited a similar dependence on the calcium concentration. We interpret these results as an indication that CabA can function properly outside the cell to yield a functional matrix, supporting the previous hypothesis that CabA is a protein released into the extracellular matrix. Taken together, the results led us to reconfirm that CabA is an extracellular matrix protein and requires calcium for its optimum activity.

### Calcium-induced conformational change and multimerization of CabA

**Fig 12:**
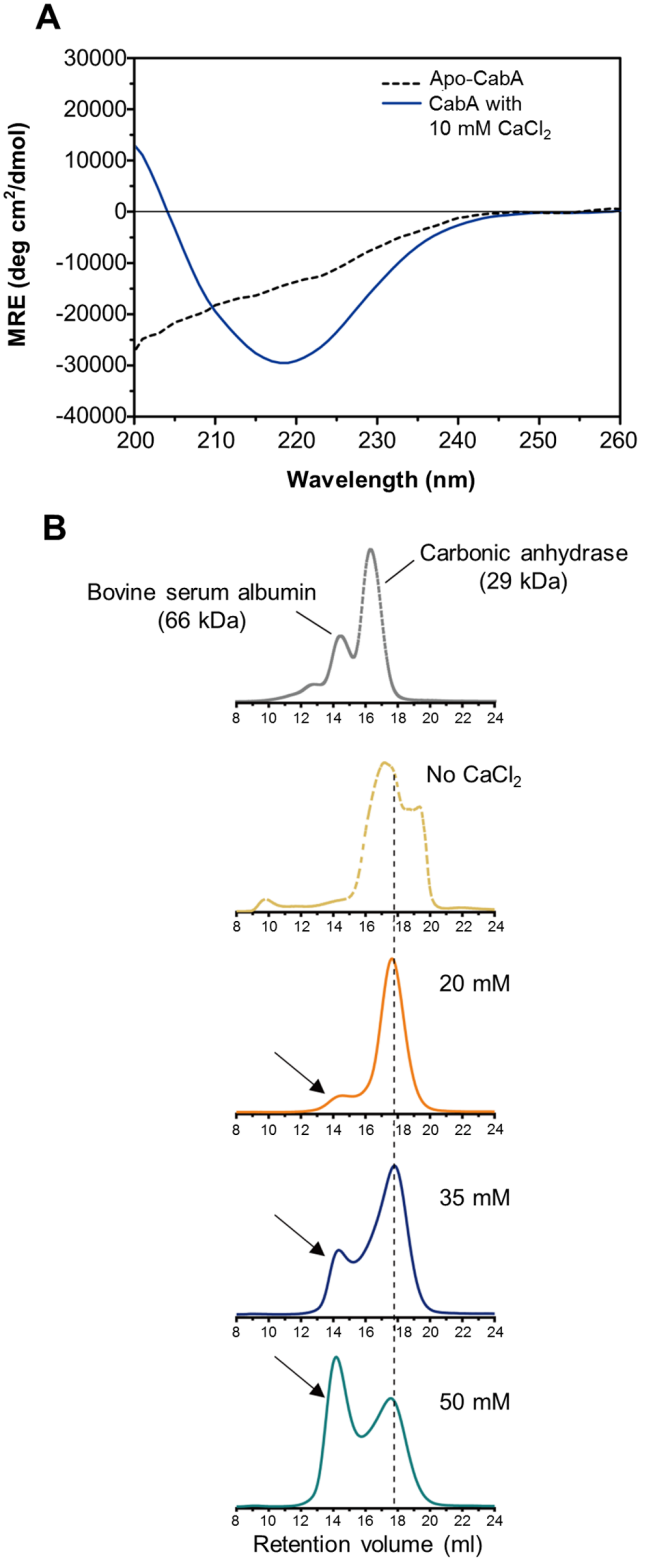
Calcium-induced conformational change and multimerization of CabA. (A) Typical far-UV CD spectra of CabA in the absence (black dotted line) and presence (blue line) of calcium. MRE, mean residue ellipticity. (B) Calcium-dependent multimerization of CabA was assessed using size-exclusion chromatography. CabA was eluted in the absence (yellow dotted line) and presence of calcium (20 mM, orange line; 35 mM, blue line; 50 mM, green line). The molecular weight standards (gray dotted line), bovine serum albumin (66 kDa) and carbonic anhydrase (29 kDa) are indicated. Elution peaks corresponding to monomeric and multimeric CabA are indicated by a black dashed line and arrows, respectively.

**Fig 13:**
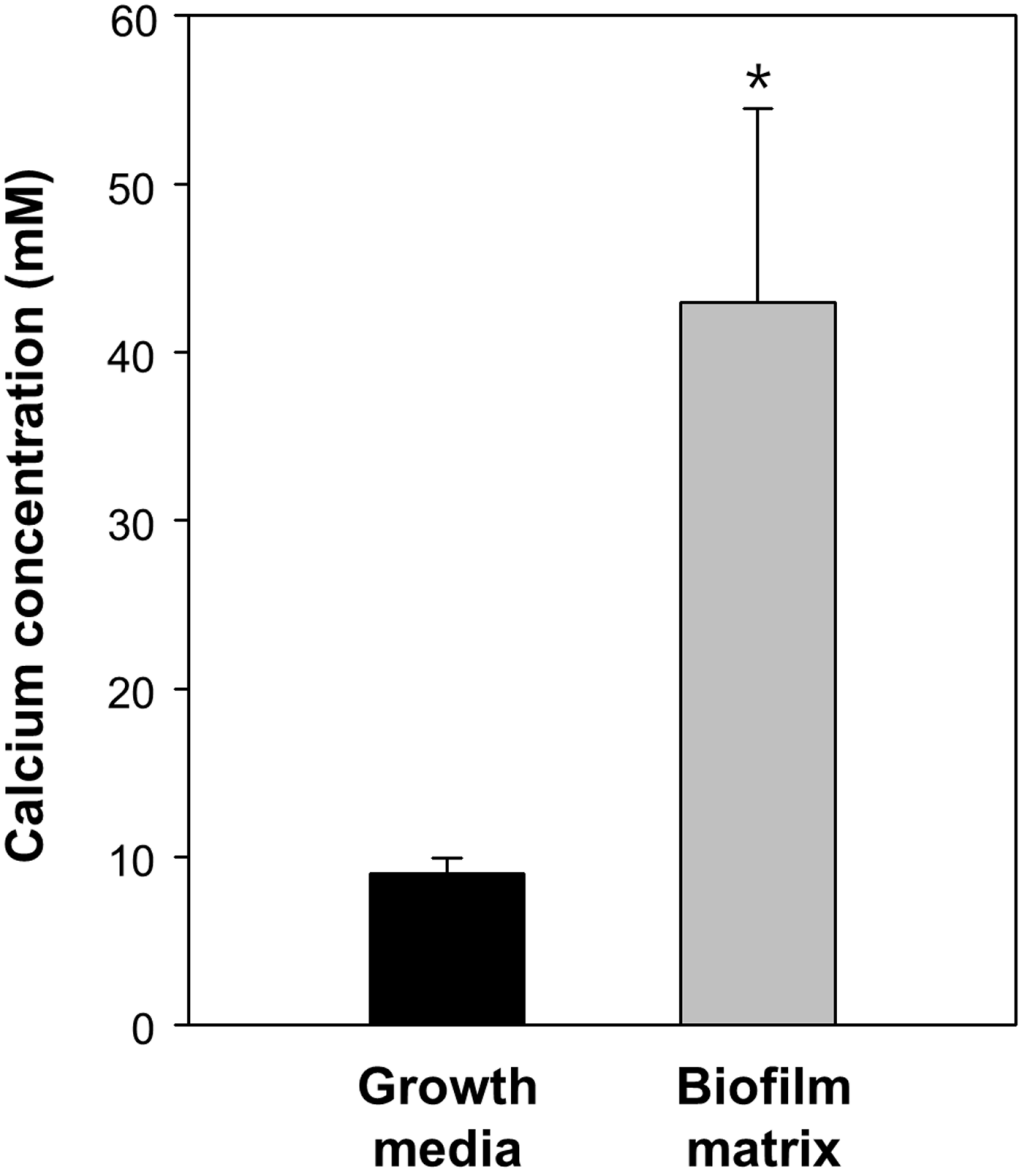
Calcium accumulation in the biofilm matrix. The JN111 Biofilm was grown in the flask containing VFMG-CF supplemented with 0.01% of arabinose and 10 mM CaCl_2_. Growth media and matrix fraction of the biofilms were prepared and the concentrations of calcium ion were measured using ICP-AES. The calcium concentrations were normalized to each volume of the matrix and growth media, and presented in molarity (mM). Error bars represent the SD. *, *P*<0.05 relative to the calcium in the biofilm growth media.

The inability of CabA to rescue the *cabA* defects in the absence of calcium (Fig 11A and 11B) postulated that calcium is probably required for the shift of CabA to a conformation functional as a biofilm matrix protein. The analysis of the far-UV circular dichroism (CD) spectrum showed that the calcium-free CabA is predominantly unfolded and lacks secondary structures (Fig 12A). However, the secondary structures of CabA were obviously formed upon addition of 10 mM calcium (Fig 12A) [38]. The properties of the CabA protein were further assessed by performing size-exclusion chromatography. The calcium-free CabA showed a broader elution pattern than the calcium-binding monomeric CabA, indicating that calcium has an important role in CabA folding (Fig 12B). A monomeric form of CabA showed a tendency to form a multimer (dimer or tetramer) in the presence of high concentration of calcium (above 20 mM) (Fig 12B). By contrast, the multimeric CabA was converted to a monomeric form by reducing calcium concentration (Fig 12B). Collectively, these results led us to hypothesize that CabA undergoes calcium-dependent conformational change and multimerization, which may be linked to the development of biofilm matrix and rugose colony. Supporting the hypothesis, the calcium in the matrix of biofilm, which was grown with 10 mM CaCl_2_ added externally, was concentrated to reach 43 mM, which is enough for induction of CabA multimerization (Fig 13).

### Extracellular complementation of the *cabA* and *brpA* mutants

**Fig 14:**
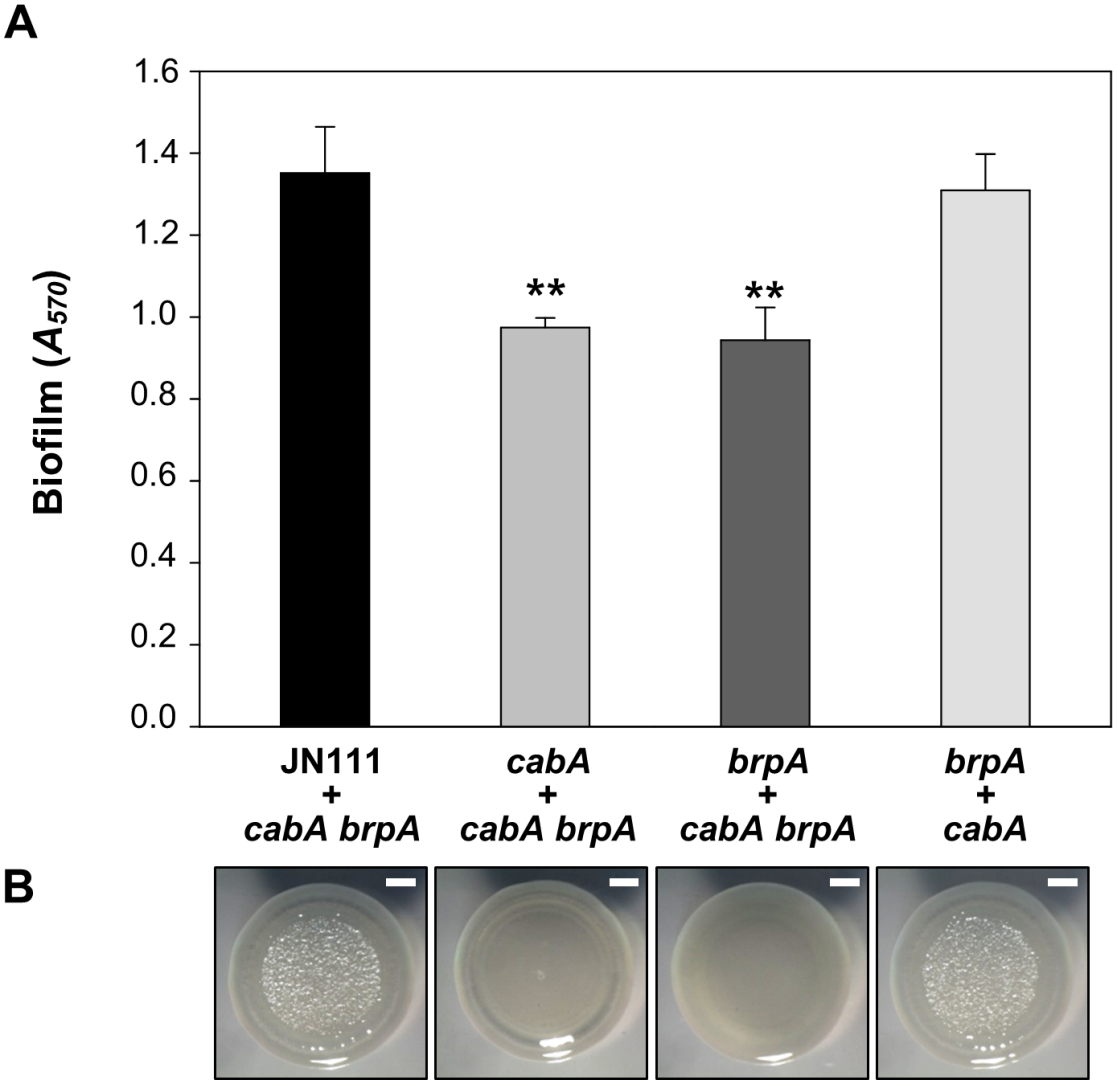
Extracellular complementation by co-culturing the mutants which lack either EPS or CabA. (A) Biofilms of the co-cultures prepared by mixing JN111, the *cabA* mutant, or the *brpA* mutant with either the *cabA brpA* double mutant or the *brpA* mutant at a 1:1 ratio were grown with VFMG-CF supplemented with 0.01% of arabinose and 10 mM of CaCl_2_ for 24 h, and quantified using CV staining. Error bars represent the SD. **, *P*<0.005 relative to the biofilm of co-culture of JN111 and the *cabA brpA* double mutant. (B) The co-cultures prepared as described above were spotted onto VFMG-CF agar plates supplemented with 0.02% of arabinose and 10 mM CaCl_2_, incubated at 30°C for 3 d, and then visualized using a stereomicroscope (Stemi DV4, Zeiss) at an 8× magnification. Bars, 1 mm; JN111, parent strain; *cabA*, *cabA* mutant; *brpA*, *brpA* mutant; *cabA brpA*, *cabA brpA* double mutant.

Since the *brp* genes (EPS-II) are known as the most important genes for EPS production and thereby biofilm formation of *V*. *vulnificus* [7,8,13,18,19], a *brpA* mutant was constructed and used to assess the EPS effects on the CabA function. Both the *brpA* and *cabA* mutants were defective in the biofilm formation when co-cultured with the *cabA brpA* double mutant which lack production of both EPS and CabA (Fig 14A). The residual biofilm levels of the *brpA* and *cabA* mutants were similar to each other and significantly lower than that of JN111 (Fig 14A). The biofilm defect of the *brpA* mutant supported the previous observation that EPS synthesized by EPS-II is an essential constituent of the *V*. *vulnificus* biofilm matrix [13] and further implied that CabA is unable to develop a functional matrix unless EPS is also present. When the *brpA* mutant was co-cultured with the *cabA* mutant, however, the resulting biofilm was similar to that of the JN111 (Fig 14A). This result indicated that CabA can extracellularly assemble an apparently functional matrix with EPS that is produced by different cells. Similarly, the smooth colony morphologies of the *brpA* and *cabA* mutants became rugose when the mutants were co-cultured (Fig 14B). The results suggested that CabA requires EPS in the matrix to perform its function for the development of mature biofilm structures and rugose colony morphology.

## Discussion

The bacterial secondary messenger, c-di-GMP, is a global regulatory molecule that controls multiple phenotype changes in the bacterial world. Among the phenotype changes affected by c-di-GMP, life style switches from free-living planktonic cells to sessile biofilms have been most extensively studied [23]. Although little is known about the c-di-GMP regulation of the phenotype changes of *V*. *vulnificus*, it has been reported that increased intracellular c-di-GMP levels induced production of EPS, a component of the *Vibrio* spp. biofilm matrix, and subsequent development of biofilm and rugose colonies [7,22]. In this study, elevated c-di-GMP induced the expression of *cabA* encoding a calcium-binding matrix protein CabA (Fig 3B and 3C). A mutation in *cabA* significantly impaired biofilm development even in the presence of elevated c-di-GMP without reducing the amount of EPS produced (Figs 4, 7, 9, and 10). The results indicated that EPS alone is not sufficient to develop the well-structured robust biofilm and that CabA is another key component of the *V*. *vulnificus* biofilm matrix.

The data presented here and bioinformatic analysis of CabA suggested that CabA is a structural protein rather than an enzymatic protein modifying or catalyzing other matrix components such as EPS to facilitate biofilm development (http://www.ncbi.nlm.nih.gov/cdd). Exogenous addition of purified CabA was able to rescue the biofilm defect of the *cabA* mutant only when calcium was available at the same time (Fig 11A). The ability of exogenously provided CabA to switch the smooth colony morphology of the *cabA* mutant to rugose disappeared when calcium was not added to the growth media (Fig 11B). These observations suggested that apo-CabA without calcium is not functional as a biofilm matrix component. Calcium-dependent multimerization of CabA, as shown in Fig 12B, indicated that the multimeric form of CabA could be a functional conformation which is competent as a biofilm matrix component. This calcium dependency of CabA is especially intriguing since calcium is a relatively abundant element in the environment of oyster, the natural niche and primary route of infection for *V*. *vulnificus* [10,39]. Therefore, it is a reasonable hypothesis that calcium is an environmental cue that multimerizes CabA to the functional conformation, rendering *V*. *vulnificus* to switch from a planktonic to a sessile life style, survive in hostile environments, and reach a concentrated infective dose when consumed by humans. Supporting the hypothesis, CabA is essential for the robust structures of the *V*. *vulnificus* biofilms on oyster shells (Fig 10).

It was noteworthy that calcium was still able to promote biofilm formation under the conditions in which *cabA* expression is limited or deficient (Figs 4C and 11A). These results suggested that calcium regulates cellular functions involved in biofilm formation besides mediating the multimerization of CabA. The previous observation that calcium alters the extracellular polysaccharides to a more biofilm-proficient phase in *V*. *vulnificus* could be a possible explanation for the calcium-dependent, yet CabA-independent, biofilm formation [40].

The majority of CabA present as a monomer under conditions of low calcium shifted to multimeric form in the presence of increasing concentrations of calcium (Fig 12B). However, the concentrations of calcium required for multimerization of CabA *in vitro* (20–50 mM) were significantly higher than those added externally to induce biofilm formation (10 mM). One possible explanation for this discrepancy is that calcium could be concentrated in the matrix by yet unknown mechanisms and the concentrations of calcium in the microenvironment where CabA multimerization occurs could be higher than those added externally for the biofilm formation experiments (Figs 4, 7, 9, 10, 11, and 14). To test this possibility, the matrix of biofilms was fractionated and the calcium concentration in the matrix was determined using ICP-AES. As shown in Fig 13, the calcium concentration in the matrix was 4.8-fold (*P*<0.05) higher than that in the biofilm growth media, indicating that calcium could be concentrated in the matrix. Consistent with this, it has been reported that excess of calcium is accumulated and immobilized within the biofilm matrices of *Pseudomonas aeruginosa* and *Enterococcus faecalis* [41,42].

There are several different mechanisms for structural proteins in the matrix to contribute to biofilm maturation [5]. Recent works demonstrated that an extracellular protein TasA forms amyloid fibers that glue cells together and provide structure to the extracellular matrix in *Bacillus subtilis* biofilms [43]. *P*. *putida* large surface-associated protein LapF, and probably *S*. *enteritidis* BapA, accumulate in certain cell surface regions and interact with each other to promote aggregations of adjacent cells, which lead to microcolony formation and subsequent biofilm maturation [44,45]. However, the amino acid sequence of CabA is not homologous to that of LapF and BapA (http://blast.ncbi.nlm.nih.gov/Blast.cgi). Furthermore, *in situ* TEM analysis revealed that functional CabA irregularly diffused into the matrix rather than retained on or concentrated along the cell envelopes (Fig 6). Another mechanism adopted by *P*. *aeruginosa* CdrA, and *V*. *cholerae* RbmC harboring carbohydrate binding domains is that the proteins bind to extracellular polysaccharides and cross-link them to polymers. Polymerization of the polysaccharides promote aggregation of cells and thus increase structural integrity of the biofilm [46,47]. Successful formation of biofilms by co-culturing the mutants defective in the synthesis of either EPS or CabA indicates that CabA requires EPS to assemble an apparently functional matrix (Fig 14). However, CabA does not harbor carbohydrate binding domains and share homology with any of the proteins in amino acid sequences (http://www.ncbi.nlm.nih.gov/cdd). Additional work is needed to clarify precise mechanisms by which CabA enable cells to build more structured biofilms.

In summary, CabA is a calcium-binding protein contributing to biofilm formation in a calcium-dependent manner and secreted via functional CabB and CabC. CabA is distributed distally from the cells throughout the biofilm matrix and produced as the *V*. *vulnificus* biofilm matures under conditions of elevated c-di-GMP. The *cabA* mutant was significantly defective in the development of rugose colony morphology and robust biofilms in flow cells and on oyster shells. Purified CabA, added exogenously along with calcium, was able to restore the *cabA* mutant abilities of forming biofilm and rugose colony morphology. Calcium binding induces conformational changes of CabA to multimeric forms and CabA can assemble a functional matrix when EPS coexist. Consequently, CabA is a calcium-binding protein functional in the extracellular matrix and essential for the development of well-structured mature biofilms and rugose colony of *V*. *vulnificus*.

## Materials and Methods

### Culture conditions, construction of JN111, and measurement of intracellular c-di-GMP

The strains and plasmids used in this study are listed in Table 1. Unless otherwise noted, the *V*. *vulnificus* strains were grown in Luria-Bertani (LB) medium supplemented with 2.0% (w/v) NaCl (LBS) at 30°C. The *V*. *fischeri* minimal medium [48] modified to omit calcium and contain glycerol (50 mM Tris-HCl, pH 7.2, 50 mM MgSO_4_, 300 mM NaCl, 10 mM KCl, 0.33 mM K_2_HPO_4_, 18.5 mM NH_4_Cl, and 32.6 mM glycerol) (VFMG-CF) was used for biofilm formation. To manipulate c-di-GMP levels in cells, *V*. *vulnificus* JN111, which carries *dcpA* encoding a DGC [22] on the chromosome under the control of arabinose-inducible promoter P_*BAD*_ [49], was constructed as depicted in S1 Fig. JN111 was used as a parent strain in this study (Table 1), and intracellular c-di-GMP levels of the *V*. *vulnificus* strains were manipulated by adding different concentrations of arabinose in the growth media. Levels of c-di-GMP in cells were measured using LC-MS as previously described [30]. Briefly, the strains grown with VFMG-CF containing 10 mM CaCl_2_ and different concentrations of arabinose was lysed with 0.6 M HClO_4_, and spin down to obtain supernatants. Measurements of c-di-GMP in the supernatants were performed using an Acuity UPLC with a Synergi 4μ Hydro RP 80A column and a C18 Guard Cartridge (Phenomenex, Torrance, CA) on a Premier XL triple-quadrupole electrospray mass spectrometer (Waters, Milford, MA).

### Microarray analysis

A Center for Disease Control (CDC) reactor (Biosurface Technologies, Bozeman, MT) [50] containing 300 ml AB medium [300 mM NaCl, 50 mM MgSO_4_, 0.2% (w/v) vitamin-free casamino acids, 10 mM potassium phosphate, 1 mM L-arginine, pH 7.5] [51] was inoculated using 3 ml of overnight culture of *V*. *vulnificus* CMCP6 and biofilms were formed for 4 d at 30°C using the procedures developed by Park and colleagues [52]. Total cellular RNAs were isolated from the biofilm cells attached on the surfaces of the coupons and the planktonic cells suspended in the culture broth using an RNeasy Mini Kit (Qiagen, Valencia, CA). Aminoallyl-cDNAs were synthesized using an Amino Allyl cDNA Labeling Kit (Ambion, Austin, TX) and were respectively labeled with Cy3 or Cy5 (Amersham Pharmacia, Uppsala, Sweden). Equal amounts of the labeled cDNAs were combined to hybridize the *V*. *vulnificus* Whole-Genome Twin-Chip [53] at 42°C for 16 h and the arrays were washed, dried, scanned, and analyzed by GenePix Pro 3.0 software (Axon Instruments, Union City, CA). The ORF spots that showed a 2.5 (that is, 1.322 in log_2_ scale)-fold or greater difference in expression with a *P* value of < 0.05 were considered to be differentially expressed in biofilms.

### RNA purification and transcript analysis

Total RNAs were isolated from JN111 grown to *A*_*600*_ of 0.6 with LBS containing 0.01% (w/v) of arabinose using an RNeasy Mini Kit (Qiagen) and used for reverse-transcription PCR (RT-PCR) analyses of the *cabABC* transcription pattern. For RT-PCR, a series of reactions was performed with Transcriptor First Strand cDNA Synthesis Kit (Roche, Mannheim, Germany) according to the manufacturer’s procedures to synthesize cDNA. PCR amplification of the cDNA was performed using standard protocols with a pair of primers, RTcabA and RTcabC2, which are designed to hybridize to the 3′ ends of *cabA* and *cabC*, respectively (S2 Table and Fig 1A). For quantitative real-time PCR (qRT-PCR) analyses of *cabA* expression, total RNAs were isolated from JN111 grown for various periods with VFMG-CF containing 10 mM CaCl_2_ and different concentrations of arabinose. cDNA was synthesized from the RNAs by using the iScript cDNA Synthesis Kit (Bio-Rad Laboratories, Hercules, CA) and real-time PCR amplification of the cDNA was performed by using the Chromo 4 real-time PCR detection system (Bio-Rad Laboratories) with a pair of primers, qRTcabA_F and qRTcabA_R (S2 Table). Relative expression levels of the *cabA* transcripts were calculated by using the 16S rRNA expression level as the internal reference for normalization.

### Purification of CabA, ICP-AES, and ITC

The coding region of *cabA* was amplified by PCR using the *V*. *vulnificus* CMCP6 chromosomal DNA and a pair of primers, cabAexp_F and cabAexp_R (S2 Table), and cloned into a His_6_ tag expression vector, pET28a(+) (Novagen, Madison, WI) to result in pYM1202 (Table 1). The His-tagged CabA was then expressed in *E*. *coli* BL21 (DE3), and purified by affinity chromatography according to manufacturer’s procedure (Qiagen). The purified CabA was dialyzed for 12 h against the storage buffer [20 mM Tris-HCl, pH 8.0, 300 mM NaCl, 0.1 mM EDTA, 0.1 mM DTT and 50% (v/v) glycerol] and kept frozen until use. The calcium-free CabA was prepared by extensively dialyzing the purified CabA against the storage buffer with EGTA (10 mM) and then subsequently against the storage buffer without EGTA. No remaining calcium in the calcium-free CabA protein was confirmed by the analysis of ICP-AES.

The purified CabA (210 μM) was digested with concentrated HNO_3_ and diluted with distilled water. The diluted CabA solution was nebulized into the plasma using MiraMist nebulizer (Burgener Research Inc., Mississauga, Canada) and the concentrations of five metal ions (Ca, Mg, Mn, Fe, and Zn) were measured using ICP-AES (Optima-4300 DV, PerkinElmer, Waltham, MA). The operating conditions developed by Cubadda and Raggi [54] were adopted and calibration was performed using the reference metal solutions as external standards.

ITC experiments were performed to determine calcium-binding parameters for CabA. For this purpose, the calcium-free CabA was reconstituted in 300 mM NaCl and 50 mM Tris-HCl, pH 8.0, and CaCl_2_ solution was prepared in the same buffer. The protein and CaCl_2_ solution were degassed by vacuum aspiration for 20 min prior to loading and titration at 25°C. The calorimetric assays were performed using a VP-ITC (MicroCal Inc., Northampton, MA). The calcium (1.2 mM) in the syringe was titrated against the 0.024 mM CabA in the reaction cell at 25°C. The stirring speed was 270 rpm, and the thermal power was recorded every 15 s. Raw data were processed and plotted using the Origin program (version 7) supplied with the instrument. The ITC experiments were repeated three times.

### Generation of the *cabA*, *cabB*, *cabC*, *brpA*, and *cabA brpA* mutants and complementation of the *cabA*, *cabB*, and *cabC* mutants

The *cabA* gene was inactivated *in vitro* by deletion (530-bp of 573-bp) of the *cabA* coding region using the PCR-mediated linker-scanning mutation method as described previously [31]. Briefly, pairs of primers cabA_F1 and cabA_R1 (for amplification of the 5’ amplicon) or cabA_F2 and cabA_R2 (for amplification of the 3’ amplicon) were designed and used as listed in S2 Table. The Δ*cabA* was amplified by PCR using the mixture of both amplicons as the template and cabA_F1 and cabA_R2 as primers. Similar experimental procedures were adopted for construction of the Δ*cabB*, Δ*cabC* and Δ*brpA in vitro*, except that primers cabB_F1, cabB_R1, cabB_F2 and cabB_R2 (for 1191-bp deleted Δ*cabB*), cabC_F1, cabC_R1, cabC_F2 and cabC_R2 (for 849-bp deleted Δ*cabC*) and brpA_F1, brpA_R1, brpA_F2, and brpA_R2 (for 448-bp deleted Δ*brpA*) were used (S2 Table). An 1.2-kb *nptI* DNA conferring resistance to kanamycin [55] was also inserted into a unique BamHI site present within the Δ*brpA* to result in Δ*brpA*::*nptI*. The resulting Δ*cabA*, Δ*cabB*, Δ*cabC*, and Δ*brpA*::*nptI* were ligated with SpeI-SphI-digested pDM4 [56] to form pYM1102, pJN1504, pJN1402, and pJN0907, respectively (Table 1). The *E*. *coli* S17-1 λ *pir*, *tra* containing either pYM1102, pJN1504, pJN1402, or pJN0907 was used as a conjugal donor to CMCP6 or JN111. The conjugation and isolation of the transconjugants were conducted as previously described [31], and resulted in the *cabA* mutants (YM112, YM112D), the *cabB* mutant (JN151D), the *cabC* mutant (JN141D), or the *brpA* mutant (JN094D), respectively (Table 1). Similarly, the *E*. *coli* S17-1 λ *pir*, *tra* containing pJN0907 was used as a conjugal donor to YM112D to construct the *cabA brpA* double mutant JN143D.

For complementation, pairs of primers, cabAcom_F and cabAcom_R, cabBcom_F and cabBcom_R or cabCcom_F and cabCcom_R, were designed and used to amplify coding regions of *cabA*, *cabB* or *cabC*, respectively (S3 Table). The amplified ORFs of *cabA*, *cabB*, and *cabC* were cloned into pJK1113 [57] under an arabinose-inducible promoter P_*BAD*_ to create pYM1109. pJN1502, and pJN1403, respectively (Table 1). The plasmids pYM1109, pJN1502, and pJN1403 were transferred into YM112D, JN151D, and JN141D, respectively, by conjugation as described above.

### Biofilm formation

Biofilms were formed using the procedure developed by O’Toole and Kolter [58] with minor modifications. Briefly, each well of the 96-well polystyrene microtiter plates (Nunc, Roskilde, Denmark) was inoculated with 200 μl of each culture diluted to an *A*_600_ 0.05 with VFMG-CF and then incubated for 24 h at 30°C without shaking. Once the planktonic cells were removed, the biofilm cells on the wall were washed with phosphate-buffered saline (PBS, pH 7.4), and then stained with 220 μl of 1% (w/v) CV solution for 15 min at room temperature. Biofilms were quantified by elution of CV with 220 μl 100% ethanol and measurement of absorbance at 570 nm (*A*_570_). When required, VFMG-CF supplemented with various amounts of CaCl_2_, arabinose, and/or calcium-free CabA was used. Similarly, inoculums of the co-cultures were prepared by mixing JN111, *cabA* mutant, or *brpA* mutant with either *cabA brpA* mutant or *brpA* mutant at a 1:1 ratio and used to form biofilms. For isolation of total RNAs and proteins from biofilms at different development stages, biofilm of JN111 was formed as described above but in a larger scale (800 μl) using 24-well polystyrene microtiter plates (SPL, Seoul, Korea).

### EPS analysis

EPS was prepared following the procedures previously described by Kim *et al*. [12]. Briefly, JN111 cells grown on LBS agar plates containing 0.02% (w/v) arabinose were suspended in PBS, and were vigorously shaken to elute EPS from cells. Cells and debris were removed by centrifugation and EPS in the supernatants was treated with RNase A (50 μg/ml), DNase I (50 μg/ ml with 10 mM MgCl_2_), and proteinase K (200 μg/ml). Subsequently, the remained polysaccharide fractions were extracted twice with phenol-chloroform, and precipitated with 2.5× volumes of 100% ethanol and resuspended in distilled water. EPS resuspensions were resolved using a 4% stacking SDS-PAGE, stained with Stains-All (Sigma-Aldrich, St. Louis, MO). The gel was subsequently destained as described previously [59], and photographed using a digital camera (PowerShot SX220 HS, Canon, Tokyo, Japan).

### Biofilm fractionation, ICP-AES, and Western blot analysis

Biofilms were formed by gentle shaking of the glass flask (Duran, Mainz, Germany) containing 100 ml VFMG-CF supplemented with 0.01% (w/v) of arabinose and 10 mM of CaCl_2_ for 8 h at 30°C. Planktonic cells were removed by rinsing gently with PBS, after which the biofilms were collected with a cell scraper (SPL) and resuspended in PBS. Biofilms were disrupted by sonication (VC130 Ultrasonic Processor, Sonics & Materials, Inc., Newtown, CT) and then fractionated into cell fractions (pellets) and matrix fractions (supernatants) by centrifugation as previously described [60]. The calcium concentrations in the biofilm matrix (equivalent to 30 μl) and growth media were determined using ICP-AES (Optima-4300 DV, PerkinElmer) as described above. For Western blot analysis, proteins (10 μg) from the lysates of cell fractions and concentrates of matrix fractions were resolved by SDS-PAGE [61]. Immunoblotting of CabA was performed using the rabbit anti-CabA antibody (AbFrontier, Seoul, Korea) as previously described [31]. Similar Western blot analyses were adopted to quantify the levels of CabA in the JN111 biofilms (that is, in the cells and matrices) grown with different concentrations of arabinose or at different biofilm development stages.

### Immunogold labeling and TEM

Immunogold labeling of CabA was performed following the procedures developed by Martinez-Gil *et al*. [45] with minor modifications. Biofilms were grown on nickel grids in 24-well polystyrene microtiter plate wells (SPL) containing VFMG-CF supplemented with 0.01% (w/v) of arabinose and 10 mM CaCl_2_ for 12 h at 30°C. The grids were treated with blocking buffer [1% (w/v) skim milk in PBS with 0.1% (v/v) Tween20], incubated for 3 h with the rabbit anti-CabA antibody (AbFrontier), and exposed to a goat anti-rabbit secondary antibody conjugated to 10-nm gold particles (Sigma-Aldrich) for 80 min. The biofilms on the grids were negatively stained with 2% (v/v) solution of uranyl acetate and visualized using TEM (JEM1010, JEOL, Tokyo, Japan) at 80 kV accelerating voltage.

### Flow cells, CSLM, and SEM

For structural analyses, biofilms of the strains were formed in flow cell chambers as described elsewhere [46]. Glass coverslips were attached on polycarbonate flow cells with individual channel dimensions of 1 × 4 × 40 mm. Each flow cell was inoculated with 100 μl of the culture diluted to *A*_600_ of 0.1, and inverted to allow bacteria to attach to the coverslip for 1 h without flow. Then VFMG-CF supplemented with 0.01% (w/v) arabinose and 10 mM CaCl_2_ was flowed at a constant rate of 8 ml/h using a Minipuls Evolution peristaltic pump (Gilson, Villiers, France) to grow biofilm for 3 d. The flow cell chambers with grown biofilms were photographed using a digital camera (PowerShot SX220 HS, Canon).

For CSLM analysis, biofilms on the coverslips were stained with LIVE/DEAD *Bac*Light Viability Kit containing SYTO9 and propidium iodide (Invitrogen) for 15 min in the dark and visualized by CSLM (LSM710, Zeiss, Jena, Germany). The biofilm images were processed using Zeiss Zen software (Zeiss). Biofilms on the coverslips were fixed, and dehydrated for SEM analysis by the procedures developed by Lim *et al*. [62]. Briefly, the biofilms were fixed using a buffer [2% (v/v) paraformaldehyde, 0.2% (v/v) glutaraldehyde, 0.1 M sodium cacodylate, pH 7.3] for 1 h at 4°C, washed with 0.05 M sodium cacodylate for 20 min at 4°C, and then postfixed with 1% (v/v) osmium tetroxide in 0.05 M sodium cacodylate (pH 7.3) for 45 min at 4°C. Fixed biofilms were washed with distilled water, dehydrated in a series of increasing concentrations of ethanol (50, 70, 80, 90, and 100%), and then dried in hexamethyldisilazane. Dried biofilms were mounted on an aluminum stub, coated with platinum using a sputter coater (BAL-TEC SCD 005, BAL-TEC AG, Balzers, Liechtenstein), and visualized using SEM (Supra 55VP, Zeiss). To determine the structure of biofilms on oyster shells, biofilms of the strains were grown for 24 h on fragmented oyster shells (1 × 1 cm) in 24-well polystyrene microtiter plates (SPL) containing VFMG-CF supplemented with 10 mM CaCl_2_ and with or without 0.01% arabinose. Biofilms on oyster shells were fixed, washed, dehydrated, dried, mounted, coated and visualized using SEM as described above.

### Colony morphology assay

For the analysis of colony morphology, 2 μl of cultures grown to *A*_600_ of 0.8 was spotted onto VFMG-CF agar plates supplemented with or without 0.02% (w/v) of arabinose and 10 mM CaCl_2_. When required, various amounts of calcium-free CabA was exogenously provided to the spotted colonies. Similarly, 2 μl of co-cultures prepared by mixing JN111, the *cabA* mutant, or the *brpA* mutant with either the *cabA brpA* double mutant or the *brpA* mutant at a 1:1 ratio were spotted as described above. The resulting colonies grown at 30°C for 3 d were visualized using a stereomicroscope (Stemi DV4, Zeiss).

### CD and size-exclusion chromatography analysis

CD spectroscopy was used to evaluate the effect of calcium on the CabA conformation. The calcium-free CabA (0.05 mg/ml) in 300 mM NaCl and 50 mM Tris-HCl, pH 8.0 was subjected to far-UV CD measurements at 25°C using a 1-mm path length quartz cuvette in a Jasco J-815 CD spectrometer (Jasco, Tokyo, Japan). The CabA protein was incubated with 10 mM CaCl_2_ for 1 h at room temperature, and its CD spectra were measured under the same conditions. CD Spectra were acquired over the wavelength range of 200–260 nm and converted into mean residue ellipticity (MRE, degree cm^2^/dmol). Blank spectra of the buffer without the protein were subtracted.

Calcium-induced conformational change of CabA was further assessed by size-exclusion chromatography using a Superdex 75 10/300 GL gel filtration column (GE Healthcare, Waukesha, WI) installed on an AKTA FPLC system (GE Healthcare). The multimeric CabA obtained with 100 mM CaCl_2_ was serially diluted to various calcium concentrations. The diluted samples were then injected onto the pre-equilibrated column with 300 mM NaCl, 20 mM Tris-HCl, pH 8.0 and corresponding concentrations of calcium and eluted at a flow rate of 0.4 ml/min at room temperature. The effluent was monitored by measuring absorbance at 280 nm. Bovine serum albumin (66 kDa) and carbonic anhydrase (29 kDa) were used as the molecular weight standards.

### Data analyses

Averages and standard deviation (SD) were calculated from at least three independent experiments. All other data were analyzed by Student's *t* tests with the SAS program (SAS software; SAS Institute Inc.). Significance of differences between experimental groups was accepted at a *P* value of < 0.05.

### Accession numbers

GenBank accession numbers for the proteins and genome sequences are as follows: DcpA, AAO08284.1; CabA, AAO08434.1; CabB, AAO08435.2; CabC, AAO08436.1; BrpA, AAO08445.1; *V*. *vulnificus* CMCP6 genome, AE016795 and AE016796. All primary microarray data were deposited in the Gene Expression Omnibus [GEO (http://www.ncbi.nlm.nih.gov/projects/geo/)] database under accession number (GSE59753).

## Supporting Information

S1 Fig Generation of JN111 which expresses *dcpA* under the P_*BAD*_ promoter. A diagram for construction of P_*BAD*_-*dcpA* fusion by insertion of the *araC*-P_*BAD*_ cassette into the upstream regulatory region of *dcpA* on the *V*. *vulnificus* CMCP6 chromosome. The promoter region and 5′-distal end of *dcpA* were amplified with pairs of primers A1 and A2 (amplicon A) or B1 and B2 (amplicon B), respectively. An NcoI restriction site was added to the 3′-end of the primer A2 and 5′-end of the primer B1, respectively. The 1.9-kb amplicon A-B containing a NcoI site was amplified using the mixture of both amplicons as the template and A1 and B2 as primers, and then ligated into pDrive (Qiagen, Valencia, CA), forming pJN1105 (Table 1). The *araC*-P_BAD_ cassette of pBAD24 [49] was amplified by PCR using primers C1 and C2. The PCR product (amplicon C) was ligated with the NcoI-digested pJN1105 to form pJN1106 (Table 1). The 3.2-kb XbaI-SphI digestion product of pJN1106 was isolated and ligated with pDM4 digested with the same enzymes, forming pJN1107. The *E*. *coli* S17-1λ*pir*, *tra* containing pJN1107 was used as conjugal donor to *V*. *vulnificus* CMCP6. A transconjugant with a genetic background P_BAD_-*dcpA* was isolated and named JN111 (Table 1). *Solid lines*, chromosomal and plasmid DNA; *large Xes*, genetic crossing over; *bent arrows*, the transcription orientation of P_*BAD*_; *closed arrows*, locations of the oligonucleotide primers used for PCR; A1, dcpA_F1; A2, dcpA_R1; B1, dcpA_F2; B2, dcpA_R2; C1, pBAD24_F; C2, pBAD24_R (S3 Table).
(PDF)

S2 Fig Effects of arabinose on biofilm formation. Each well of the 96-well microtiter plates (Nunc, Roskilde, Denmark), containing VFMG-CF supplemented with 10 mM of CaCl_2_ and various levels of arabinose as indicated, was inoculated with 200 μl of the JN111 culture diluted to an *A*_600_ 0.05. The microtiter plates were incubated for 24 h at 30°C without shaking. Once planktonic cells were removed, the biofilm cells on the wall were washed with PBS, and then stained with 220 μl of 1% crystal violet (CV) solution for 15 min at room temperature. Biofilms were quantified by elution of CV with 220 μl 100% ethanol and measurement of absorbance at 570 nm (*A*_570_). Error bars represent the SD.
(PDF)

S3 Fig Visualization of crystal violet-stained biofilms in test tubes. Each 14 ml round-bottom test tubes (BD Biosciences, Erembodegem, Belgium), containing VFMG-CF supplemented with (A) or without (B) 0.01% arabinose and with various levels of CaCl_2_ as indicated, was inoculated with 1 ml of each culture diluted to an *A*_600_ 0.05. The tubes were incubated for 24 h at 30°C without shaking. Once the planktonic cells were removed, the biofilms on the wall were washed with PBS, and then stained with 1.2 ml of 1% CV solution for 15 min at room temperature. CV-stained biofilms were washed with distilled water and photographed using a digital camera (PowerShot SX220 HS, Canon, Tokyo, Japan). JN111, parent strain; *cabA*, *cabA* mutant.
(PDF)

S1 Table Genes and their products of which expression is differentially regulated in biofilm cells versus planktonic cells. (PDF)

S2 Table Preferential expression of *cabABC* in biofilms. (PDF)

S3 Table Oligonucleotides used in this study. (PDF)
