# Correction: The *cabABC* Operon Essential for Biofilm and Rugose Colony Development in *Vibrio vulnificus*

**DOI:** 10.1371/journal.ppat.1005252

**Published:** 2015-10-23

**Authors:** Jin Hwan Park, Youmi Jo, Song Yee Jang, Haenaem Kwon, Yasuhiko Irie, Matthew R. Parsek, Myung Hee Kim, Sang Ho Choi

There are errors in the Funding section. The correct funding information is as follows:

This work was supported by the Mid-career Researcher Program Grants (2015R1A2A1A13001654, to SHC) and (2014R1A2A1A01005971, to MHK) through National Research Foundation (NRF) funded by the Ministry of Science, ICT, and Future Planning; the Ministry of Food and Drug Safety Grant (14162MFDS972, to SHC) in 2015; and the R&D Convergence Center Support Program of the Ministry of Agriculture, Food and Rural Affairs (to SHC), Republic of Korea. MRP was supported by the National Institutes of Health (R01 AI077628-05A) and YI was a University of Washington-Cystic Fibrosis Foundation Research and Development Program (UW CFF RDP) Fellow. The funders had no role in study design, data collection and analysis, decision to publish, or preparation of the manuscript.
