# Supplementary material for: The cabABC Operon Essential for Biofilm and Rugose Colony Development in Vibrio vulnificus
Source: PLoS Pathog. 2015 Sep 25;11(9):e1005192. doi: 10.1371/journal.ppat.1005192 (PMC4584020; doi:10.1371/journal.ppat.1005192)
Supplement: S1 Fig — A diagram for construction of PBAD-dcpA fusion by insertion of the araC-PBAD cassette into the upstream regulatory region of dcpA on the V. vulnificus CMCP6 chromosome. The promoter region and 5′-distal end of dcpA were amplified with pairs of primers A1 and A2 (amplicon A) or B1 and B2 (amplicon B), respectively. An NcoI restriction site was added to the 3′-end of the primer A2 and 5′-end of the primer B1, respectively. The 1.9-kb amplicon A-B containing a NcoI site was amplified using the mixture of both amplicons as the template and A1 and B2 as primers, and then ligated into pDrive (Qiagen, Valencia, CA), forming pJN1105 (Table 1). The araC-PBAD cassette of pBAD24 [49] was amplified by PCR using primers C1 and C2. The PCR product (amplicon C) was ligated with the NcoI-digested pJN1105 to form pJN1106 (Table 1). The 3.2-kb XbaI-SphI digestion product of pJN1106 was isolated and ligated with pDM4 digested with the same enzymes, forming pJN1107. The E. coli S17-1λpir, tra containing pJN1107 was used as conjugal donor to V. vulnificus CMCP6. A transconjugant with a genetic background PBAD-dcpA was isolated and named JN111 (Table 1). Solid lines, chromosomal and plasmid DNA; large Xes, genetic crossing over; bent arrows, the transcription orientation of PBAD; closed arrows, locations of the oligonucleotide primers used for PCR; A1, dcpA_F1; A2, dcpA_R1; B1, dcpA_F2; B2, dcpA_R2; C1, pBAD24_F; C2, pBAD24_R (S3 Table). (PDF) [file ppat.1005192.s001.pdf]

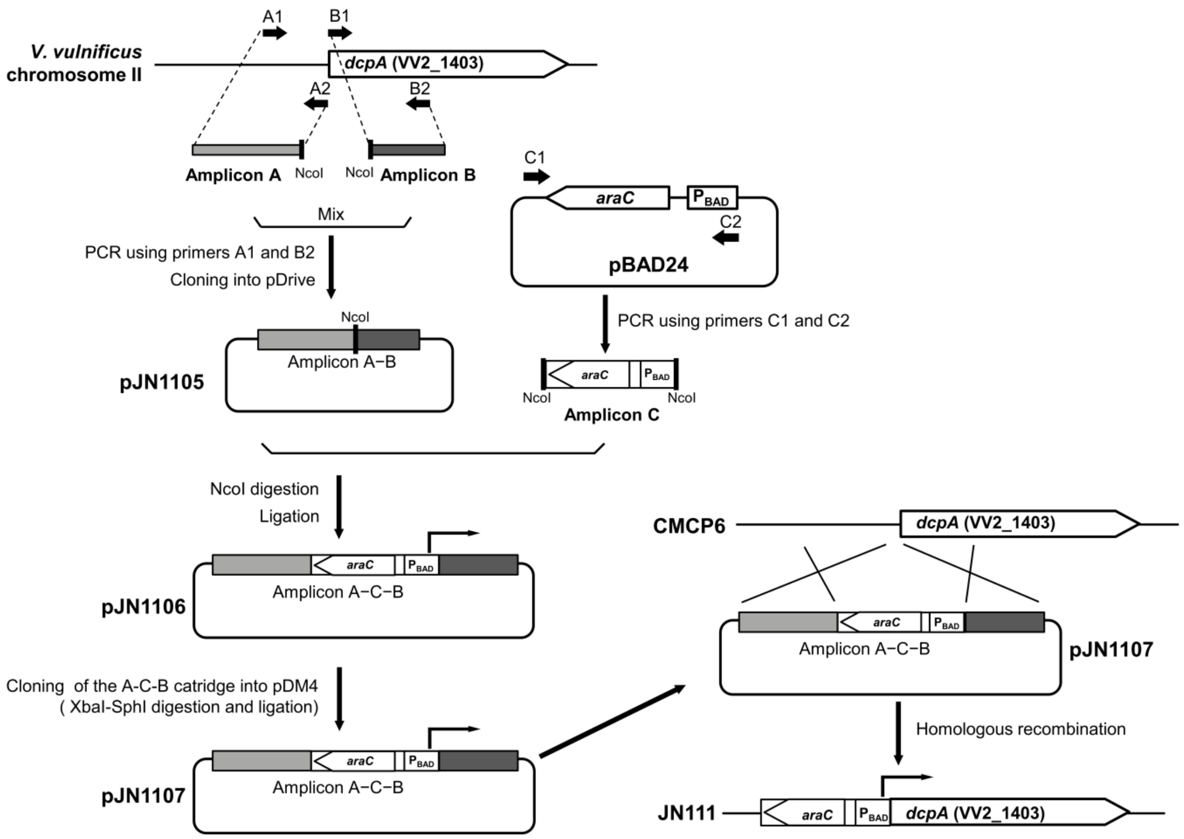

S1 Fig. Generation of JN111 which expresses *dcpA* under the P*BAD* promoter. A diagram for construction of P*BAD*-*dcpA* fusion by insertion of the *araC*-P*BAD* cassette into the upstream regulatory region of *dcpA* on the *V. vulnificus* CMCP6 chromosome. The promoter region and 5′-distal end of *dcpA* were amplified with pairs of primers A1 and A2 (amplicon A) or B1 and B2 (amplicon B), respectively. An NcoI restriction site was added to the 3′-end of the primer A2 and 5′-end of the primer B1, respectively. The 1.9-kb amplicon A-B containing a NcoI site was amplified using the mixture of both amplicons as the template and A1 and B2 as primers, and then ligated into pDrive (Qiagen, Valencia, CA), forming pJN1105 (Table 1). The *araC*-PBAD cassette of pBAD24 (Guzman *et al.*, 1995) was amplified by PCR using primers C1 and C2. The PCR product (amplicon C) was ligated with the NcoI-digested pJN1105 to form pJN1106 (Table 1). The 3.2-kb XbaI-SphI digestion product of pJN1106 was isolated and ligated with pDM4 digested with the same enzymes, forming pJN1107. The *E. coli* S17-1λ*pir*,

tra containing pJN1107 was used as conjugal donor to *V. vulnificus* CMCP6. A transconjugant with a genetic background P_{BAD}-*dcpA* was isolated and named JN111 (Table 1). *Solid lines*, chromosomal and plasmid DNA; *large Xes*, genetic crossing over; *bent arrows*, the transcription orientation of P_{BAD}; *closed arrows*, locations of the oligonucleotide primers used for PCR; A1, dcpA_F1; A2, dcpA_R1; B1, dcpA_F2; B2, dcpA_R2; C1, pBAD24_F; C2, pBAD24_R (S3 Table).

Reference

Guzman L, Dominique B, Michael JC, Beckwith J. Tight regulation, modulation, and high-level expression by vectors containing the arabinose P_{BAD} promoter. J Bacteriol. 1995;177: 4121-4130.
