# Supplementary material for: The cabABC Operon Essential for Biofilm and Rugose Colony Development in Vibrio vulnificus
Source: PLoS Pathog. 2015 Sep 25;11(9):e1005192. doi: 10.1371/journal.ppat.1005192 (PMC4584020; doi:10.1371/journal.ppat.1005192)
Supplement: S2 Fig — Each well of the 96-well microtiter plates (Nunc, Roskilde, Denmark), containing VFMG-CF supplemented with 10 mM of CaCl2 and various levels of arabinose as indicated, was inoculated with 200 μl of the JN111 culture diluted to an A 600 0.05. The microtiter plates were incubated for 24 h at 30°C without shaking. Once planktonic cells were removed, the biofilm cells on the wall were washed with PBS, and then stained with 220 μl of 1% crystal violet (CV) solution for 15 min at room temperature. Biofilms were quantified by elution of CV with 220 μl 100% ethanol and measurement of absorbance at 570 nm (A 570). Error bars represent the SD. (PDF) [file ppat.1005192.s002.pdf]

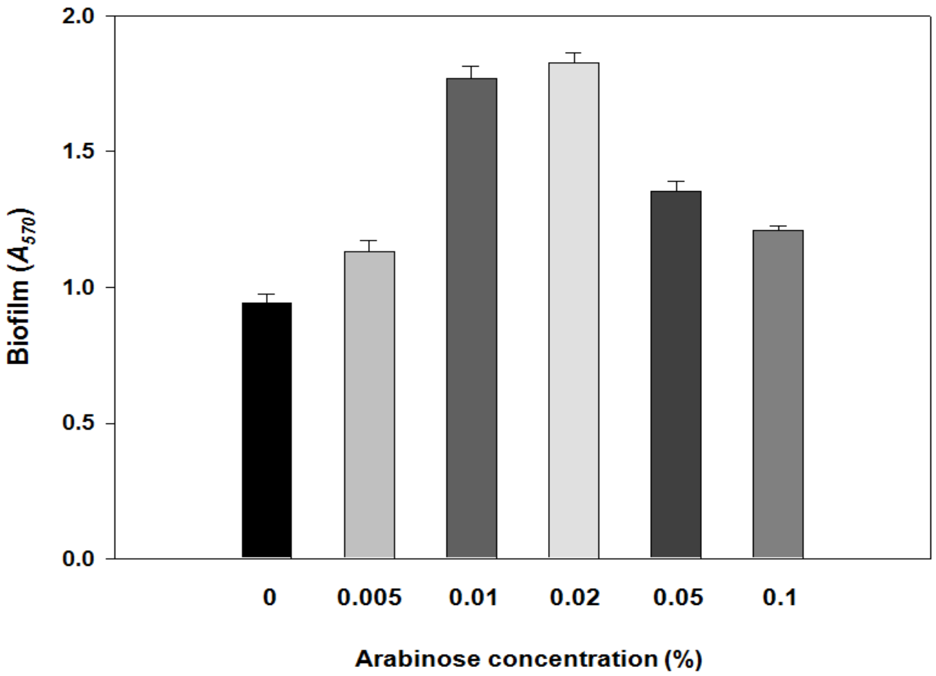

S2 Fig. Effects of arabinose on biofilm formation. Each well of 96-well microtiter plates (Nunc, Roskilde, Denmark), containing VFMG-CF supplemented with 10 mM of $CaCl_2$ and various levels of arabinose as indicated, was inoculated with 200 μl of the JN111 culture diluted to an A_{600} 0.05. The microtiter plates were incubated for 24 h at 30°C without shaking. Once planktonic cells were removed, the biofilm cells on the wall were washed with PBS, and then stained with 220 μl of 1% crystal violet (CV) solution for 15 min at room temperature. Biofilms were quantified by elution of CV with 220 μl 100% ethanol and measurement of absorbance at 570 nm (A_{570}). Error bars represent the SD.
