# Supplementary material for: The cabABC Operon Essential for Biofilm and Rugose Colony Development in Vibrio vulnificus
Source: PLoS Pathog. 2015 Sep 25;11(9):e1005192. doi: 10.1371/journal.ppat.1005192 (PMC4584020; doi:10.1371/journal.ppat.1005192)
Supplement: S3 Fig — Each 14 ml round-bottom test tubes (BD Biosciences, Erembodegem, Belgium), containing VFMG-CF supplemented with (A) or without (B) 0.01% arabinose and with various levels of CaCl2 as indicated, was inoculated with 1 ml of each culture diluted to an A 600 0.05. The tubes were incubated for 24 h at 30°C without shaking. Once the planktonic cells were removed, the biofilms on the wall were washed with PBS, and then stained with 1.2 ml of 1% CV solution for 15 min at room temperature. CV-stained biofilms were washed with distilled water and photographed using a digital camera (PowerShot SX220 HS, Canon, Tokyo, Japan). JN111, parent strain; cabA, cabA mutant. (PDF) [file ppat.1005192.s003.pdf]

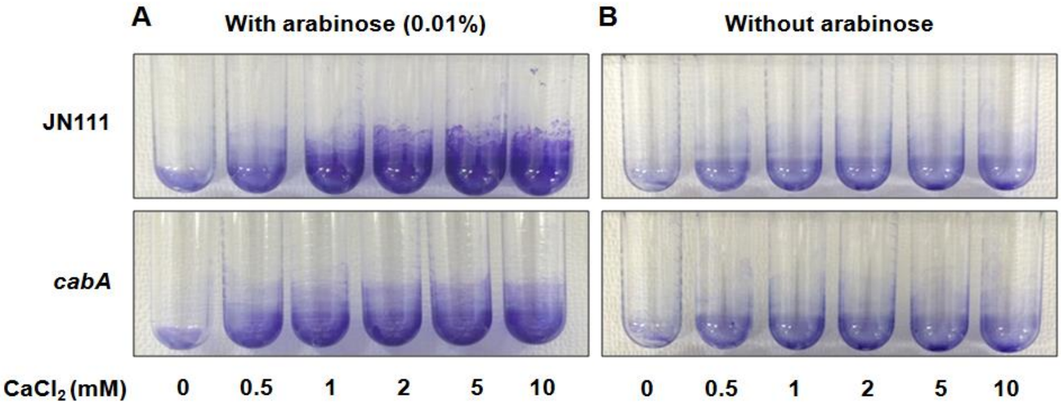

S3 Fig. Visualization of CV-stained biofilms in test tubes. Each 14 ml round-bottom test tubes (BD Biosciences, Erembrodegem, Belgium), containing VFMG-CF supplemented with (A) or without (B) 0.01% arabinose and with various levels of $CaCl_2$ as indicated, was inoculated with 1 ml of each culture diluted to an A_{600} 0.05. The tubes were incubated for 24 h at 30°C without shaking. Once the planktonic cells were removed, the biofilms on the wall were washed with PBS, and then stained with 1.2 ml of 1% CV solution for 15 min at room temperature. CV-stained biofilms were washed with distilled water and photographed using a digital camera (PowerShot SX220 HS, Canon, Tokyo, Japan). JN111, parent strain; *cabA*, *cabA* mutant.
