# Supplementary material for: The cabABC Operon Essential for Biofilm and Rugose Colony Development in Vibrio vulnificus
Source: PLoS Pathog. 2015 Sep 25;11(9):e1005192. doi: 10.1371/journal.ppat.1005192 (PMC4584020; doi:10.1371/journal.ppat.1005192)
Supplement: S1 Table — (PDF) [file ppat.1005192.s004.pdf]

S1 Table. Genes and their products of which expression is differentially regulated in biofilm cells versus planktonic cells[a]

UP-REGULATED		
**Locus tag**	**M value[b]**	**Gene product**
VV1_0028	3.521	TRAP-type C4-dicarboxylate transport system, large permease protein
VV1_0029	5.399	TRAP-type C4-dicarboxylate transport system, small permease protein
VV1_0030	6.098	TRAP-type C4-dicarboxylate transport system, periplasmic protein
VV1_0336	1.938	Predicted deacylase
VV1_0403	1.490	Putative hemolysin
VV1_0404	1.565	Conserved hypothetical protein
VV1_0405	2.012	Hypothetical protein
VV1_0406	1.419	Hypothetical protein
VV1_0407	1.381	Hypothetical protein
VV1_0408	1.381	Uncharacterized membrane-associated protein
VV1_0411	1.490	Choline-glycine betaine transporter
VV1_0413	1.438	D-amino acid dehydrogenase, small subunit
VV1_0436	1.502	HesB family protein
VV1_0437	2.114	NifU homolog involved in Fe-S cluster formation
VV1_0438	2.041	Aminotransferase NifS, class V
VV1_0439	1.486	Predicted transcriptional regulator
VV1_0450	3.766	Malate synthase A
VV1_0463	1.322	Phosphate ABC transporter, permease protein
VV1_0501	1.932	Carbon starvation protein, putative membrane protein
VV1_0842	2.548	Putative outer membrane receptor protein, mostly Fe transport
VV1_0843	1.960	Hypothetical protein
VV1_0950	2.329	Putative integral membrane protein
VV1_0981	3.293	Fatty oxidation complex, alpha subunit
VV1_0982	2.322	Fatty oxidation complex, beta subunit
VV1_0985	2.139	Hypothetical protein
VV1_1229	3.047	Predicted transcriptional regulator, MerR family
VV1_1236	1.960	3-dehydroquinate dehydratase II
VV1_1237	5.123	Acetyl-coenzyme A synthetase
VV1_1238	2.000	Putative DNA polymerase III, epsilon subunit
VV1_1239	2.224	Predicted signal-transduction protein containing cAMP-binding and CBS domains
VV1_1242	3.083	Sensor histidine kinaseresponse regulator containing sodiumprotline symporter
VV1_1243	4.198	Sodiumsolute symporter family protein
VV1_1244	4.741	Predicted membrane prtoein
VV1_1370	1.322	Acetylornithine deacetylase
VV1_1371	2.540	N-acetyl-gamma-glutamyl-phosphate reductase
VV1_1372	2.699	Acetylglutamate kinase
VV1_1373	2.977	Argininosuccinate synthase
VV1_1374	2.373	Bifunctional protein ArgH
VV1_1466	2.108	Ornithine carbamoyltransferase
VV1_1588	1.381	RNA polymerase sigma-38 factor RpoS
VV1_1753	1.527	Hypothetical protein

VV1_1800	1.608	Hypothetical protein
VV1_1835	2.178	Hypothetical protein
VV1_1836	1.801	Hypothetical protein
VV1_1837	2.816	Conserved hypothetical protein
VV1_1840	1.494	50S ribosomal protein L31 type B
VV1_1841	1.626	Putative p-aminobenzoyl-glutamate transporter
VV1_1846	1.791	FabH, 3-oxoacyl-
VV1_1896	1.983	Oxidoreductase, acyl-CoA dehydrogenase family
VV1_1975	2.191	Fatty oxidation complex, beta subunit
VV1_2090	1.370	SpoVR-like protein
VV1_2091	1.569	Conserved hypothetical protein
VV1_2092	1.837	Purtative Ser protein kinase
VV1_2224	2.388	Uncharacterized ABC transporter, periplasmic protein
VV1_2237	4.430	Extracellular solute-binding protein, family 7
VV1_2238	2.565	TRAP-type mannitolchloroaromatic compound transport system, small permease component
VV1_2239	1.676	TRAP-type mannitolchloroaromatic compound transport system, large permease component
VV1_2268	3.252	Putative ATP-binding protein
VV1_2269	2.565	Putative membrane protein
VV1_2270	1.868	Transcriptional regulator TyrR
VV1_2376	2.095	Conserved hypothetical protein
VV1_2652	1.502	Hypothetical protein
VV1_2678	1.680	Lactoylglutathione lyase-related enzyme
VV1_2687	1.322	Hypothetical protein
VV1_2696	1.527	Hypothetical protein
VV1_2703	1.662	Amino acid ABC transporter, periplasmic amino acid-binding protein
VV1_2722	2.178	Predicted hydrolase of the HAD superfamily
VV1_2723	2.152	Methyl-accepting chemotaxis protein
VV1_2724	2.293	Hypothetical protein
VV1_2725	1.756	Trypsin-like serine protease
VV1_2728	1.761	PrpE protein
VV1_2729	2.396	Conserved hypothetical protein
VV1_2730	3.095	Aconitate hydratase 1
VV1_2731	3.926	Methylcitrate synthase
VV1_2732	4.252	Carboxyphosphonoenolpyruvate phosphonomutase
VV1_2733	4.128	Transcriptional regulator, GntR family
VV1_2734	1.900	Bacterial periplasmic substrate-binding protein
VV1_2735	1.989	Conserved hypothetical protein
VV1_2737	1.927	Hypothetical protein
VV1_2739	1.358	Conserved hypothetical protein
VV1_2766	1.535	2-keto-4-pentenoate hydratase2-oxohepta-3-ene-1,7-dioic acid hydratase (catechol pathway)
VV1_2767	2.366	Homogentisate 1,2-dioxygenase
VV1_2768	2.427	Hemolysin VllY
VV1_2772	1.565	Homoserine O-succinyltransferase
VV1_2831	2.373	Oxidoreductase, acyl-CoA dehydrogenase family
VV1_2832	2.336	Transcriptional regulator, TetR family
VV1_2846	3.444	Predicted membrane protein
VV1_2847	2.059	DNA-binding response regulator, OmpR family
VV1_2848	1.816	Sensor histidine kinase

VV1_2856	2.482	Hypothetical protein
VV1_2936	1.704	Putative transcriptional regulator
VV1_2939	2.690	Hypothetical protein
VV1_2963	1.494	Conserved hypothetical protein
VV1_2968	1.515	Putative iron-regulated protein A
VV1_3065	1.431	Anthranilate synthase component II
VV1_3067	1.502	Indole-3-glycerol phosphate synthase IgpSphosphoribosylanthranilate isomerase TrpF
VV1_3132	2.506	2,4-dienoyl-CoA reductase
VV1_3174	2.071	Putative aspartate aminotransferase
VV2_0055	1.404	MoxR-like ATPase
VV2_0078	1.355	Response regulator containing a CheY-like receiver domain and an HD-GYP domain
VV2_0126	2.204	Hypothetical protein
VV2_0196	1.358	Opacity protein and related surface antigen
VV2_0205	1.523	EAL domain protein
VV2_0230	1.821	Cytochrome B561
VV2_0231	1.419	Conserved hypothetical protein
VV2_0237	1.490	Putative 5'-nucleotidase
VV2_0248	2.662	Putative CHO cell elongating factor
VV2_0249	3.047	Long-chain fatty acid transport protein
VV2_0264	1.671	Predicted signal transduction protein containing a membrane domain, an EAL and a GGDEF domain
VV2_0331	2.776	Conserved hypothetical protein
VV2_0349	1.340	3-Oxoacyl-(acyl-carrier-protein) synthase III
VV2_0381	1.427	Putative arginase family protein
VV2_0388	1.766	Hypothetical protein
VV2_0403	1.900	Cytolysin secretion protein VvhB
VV2_0404	1.894	Hemolysincytolysin VvhA
VV2_0454	1.727	Pterin-4-alpha-carbinolamine dehydratase
VV2_0455	2.474	Phenylalanine-4-hydroxylase
VV2_0456	2.035	Acetyl-CoA synthase
VV2_0492	1.599	Acyl-CoA dehydrogenase
VV2_0493	1.569	NAD-dependent aldehyde dehydrogenase
VV2_0494	1.653	Acetyl-CoA acetyltransferase
VV2_0495	1.776	Predicted transcriptional regulator, MerR family
VV2_0496	1.653	Acyl-CoA dehydrogenase
VV2_0497	1.407	Acetyl-CoA carboxylase, carboxyltransferase component
VV2_0550	1.613	Transcriptional regulator, SorC family
VV2_0552	1.894	Transaldolase B
VV2_0558	2.071	3-deoxy-D-arabino-heptulosonate 7-phosphate (DAHP) synthase
VV2_0559	2.396	Outer membrane protein, OmpA family
VV2_0560	1.884	Polyprenyltransferase (cytochrome oxidase assembly factor)
VV2_0579	1.404	Putative thioredoxin
VV2_0654	1.336	Transposase and inactivated derivative
VV2_0680	1.955	Putative permease
VV2_0681	2.245	Hypothetical protein
VV2_0717	2.041	Hypothetical protein
VV2_0766	2.279	Hypothetical protein
VV2_0814	1.466	Putative lipoprotein
VV2_0816	3.059	Arginine ABC transporter, periplasmic arginine-binding protein

VV2_0817	2.671	Arginine ABC transporter, permease protein
VV2_0818	2.653	Arginine ABC transporter, permease protein
VV2_0819	1.604	Pyridoxamine 5'-phosphate oxidase
VV2_0839	1.771	Chorismate mutase
VV2_0870	1.704	Sigma 54-dependent transcriptional regulator
VV2_0891	1.427	Conserved hypothetical protein
VV2_0919	1.617	Conserved hypothetical protein
VV2_0947	2.816	Hypothetical protein
VV2_0989	1.527	Alkyl sulfatase and related hydrolase
VV2_1011	2.000	Hydroxamate-dependent iron ABC transporter, ATP-binding protein
VV2_1012	1.569	Hydroxamate-dependent iron ABC transporter, periplasmic protein
VV2_1037	1.544	PilB-related protein
VV2_1137	2.245	Conserved hypothetical protein
VV2_1166	1.523	Chemotaxis signal transduction protein CheW
VV2_1168	1.355	Chemotaxis protein histidine kinase CheA
VV2_1169	1.667	Chemotaxis protein CheY
VV2_1170	1.582	Putative anti-anti-sigma regulatory factor
VV2_1203	1.868	Conserved hypothetical protein
VV2_1204	1.411	Glutathione synthase
VV2_1210	1.523	Hypothetical protien
VV2_1215	1.771	Putative transcriptional regulator, TetR family
VV2_1216	1.595	Putative hydrolase
VV2_1279	1.362	GGDEF family protein
VV2_1458	1.878	Spermidineputrescine ABC transporter, permidineputrescine-binding periplasmic protein
VV2_1468	1.949	Conserved hypothetical protein
VV2_1471	1.680	CuZn superoxide dismutase
VV2_1472	1.868	AnkB protein
VV2_1479	1.454	Putative permease
VV2_1570	3.108	Transcriptional regulator, BrpT
VV2_1571	2.766	A protein with putative calcium-binding motifs
VV2_1572	1.544	ABC-type transporter
VV2_1573	1.411	Membrane fusion protein (MFP)
VV2_1576	1.842	Glycosyltransferase, BrpI
VV2_1577	1.599	Hypothetical protein, BrpH
VV2_1579	1.586	Putative exopolysaccharide biosynthesis protein, BrpD
VV2_1580	1.971	Putative polysaccharide export-related protein, BrpC
VV2_1582	1.470	Undecaprenyl-phosphate glucose phosphotransferase, BrpA
VV2_1619	1.478	Putative chemotaxis protein CheV
VV2_1626	2.120	Predicted membrane protein
VV2_1657	1.556	Hypothetical protein

DOWN-REGULATED

Locus tag	M value[b]	Gene product
VV1_0053	-1.508	Histidine ammonia-lyase
VV1_0154	-1.635	Succinyl-CoA synthetase, alpha subunit
VV1_0155	-1.439	Succinyl-CoA synthetase, beta subunit
VV1_0156	-1.643	2-oxoglutarate dehydrogenase complex, E2 component, dihydrolipoamide succinyltransferase
VV1_0213	-1.618	Polar flagellin FlaE

VV1_0214	-1.934	Polar flagellin FlaD
VV1_0288	-1.525	Trehalose-6-phosphate hydrolase
VV1_0289	-1.676	PTS system, trehalose-specific IIBC component
VV1_0346	-1.338	Putative p-aminobenzoyl-glutamate transporter
VV1_0362	-1.515	Hypothetical protein
VV1_0363	-3.598	Sodiumglutamate symporter GltP
VV1_0423	-1.398	GTP binding protein EngA
VV1_0486	-1.423	Ribosome-associated inhibitor A
VV1_0542	-2.347	Acid-induced glycyl radical enzyme
VV1_0578	-1.613	UDP-N-acetylglucosamine-N-acetylmuramyl-(pentapeptide) pyrophosphoryl-undecaprenol N-acetylglucosamine transferase
VV1_0580	-3.578	UDP-N-acetylmuramoylalanine-D-glutamate ligase
VV1_0630	-1.325	Conserved hypothetical protein
VV1_0632	-1.494	RNA polymerase sigma-70 factor RpoD
VV1_0679	-1.450	UDP-N-acetylglucosamine enolpyruvyl transferase
VV1_0707	-1.362	Fructose-1,6-bisphosphatase
VV1_0743	-1.547	Ribosomal protein S5
VV1_0744	-1.633	Ribosomal protein L18
VV1_0745	-1.669	Ribosomal protein L6
VV1_0746	-1.952	Ribosomal protein S8
VV1_0747	-1.523	Ribosomal protein S14
VV1_0749	-1.787	Ribosomal protein L5
VV1_0750	-1.746	Ribosomal protein L24
VV1_0751	-1.983	Ribosomal protein L14
VV1_0753	-1.774	Ribosomal protein S17
VV1_0761	-1.665	50S ribosomal protein L4
VV1_0762	-1.786	50S ribosomal protein L3
VV1_0763	-2.151	30S ribosomal protein S10
VV1_0774	-1.383	Nucleotide sugar dehydrogenase
VV1_0776	-1.630	Putative galactosyl-transferase
VV1_0777	-1.805	Nucleoside-diphosphate-sugar epimerase
VV1_0778	-1.380	Glycosyl transferases group1 family protein
VV1_0779	-2.035	UDP-N-acetyl-D-mannosaminuronate dehydrogenase
VV1_0780	-2.009	UDP-N-acetylglucosamine 2-epimerase
VV1_0781	-1.745	Putative tyrosine-protein kinase Wzc
VV1_0782	-1.734	Hypothetical protein
VV1_0784	-1.543	Hypothetical protein
VV1_0785	-1.387	Hypothetical protein
VV1_0875	-1.344	Type II secretory pathway, component EpsF
VV1_0997	-1.427	DNA replication and repair protein RecF
VV1_1005	-1.575	Ribonuclease P protein component (RNaseP protein)
VV1_1014	-1.774	ATP synthase protein I
VV1_1019	-1.437	ATP synthase F1, alpha subunit
VV1_1196	-1.437	Putative acetyltransferase
VV1_1197	-1.532	UDP-N-acetylenolpyruvoylglucosamine reductase
VV1_1200	-1.373	Pantothenate kinase
VV1_1205	-1.669	Preprotein translocase subunit SecE
VV1_1206	-1.844	Transcription antitermination protein NusG
VV1_1207	-2.077	50S ribosomal protein L11
VV1_1208	-2.010	50S ribosomal protein L1
VV1_1209	-1.502	50S ribosomal protein L10

VV1_1338	-1.623	Translation elongation factor G (EF-G)
VV1_1360	-1.334	50S ribosomal protein L31
VV1_1387	-1.539	30S ribosomal protein S6
VV1_1442	-1.377	MSHA pilin protein MshB
VV1_1444	-1.323	Putative MSHA pilin protein MshC
VV1_1445	-1.490	Putative MSHA pilin protein MshD
VV1_1454	-1.543	Predicted membrane protein
VV1_1615	-1.440	30S ribosomal protein S16
VV1_1616	-1.375	16S rRNA processing protein RimM
VV1_1617	-1.504	tRNA-(guanine-N1)-methyltransferase
VV1_1618	-1.375	50S ribosomal protein L19
VV1_1629	-1.891	Pyruvate dehydrogenase complex repressor
VV1_1631	-1.768	Pyruvate dehydrogenase complex E2 component, dihydrolipoamide acyltransferase
VV1_1691	-1.569	Dihydropteroate synthase
VV1_1694	-1.886	Conserved hypothetical protein
VV1_1695	-1.804	N utilization substance protein A
VV1_1696	-1.560	Translation initiation factor IF-2
VV1_1697	-1.661	Ribosome-binding factor A
VV1_1727	-1.593	Phosphopentomutase
VV1_1785	-1.660	Aerobic glycerol-3-phosphate dehydrogenase
VV1_1866	-1.351	1-deoxy-D-xylulose 5-phosphate reductoisomerase
VV1_1923	-1.347	Polar flagellin FlaF
VV1_1926	-2.013	Polar flagellin FlaA
VV1_1956	-1.563	Chemotaxis responser regulator CheB
VV1_1957	-1.594	SOJ-like and chromosome partitioning protein
VV1_1958	-2.049	Chemotaxis signal transduction protein CheW
VV1_1959	-1.473	Chemotaxis signal transduction protein CheW
VV1_1960	-1.944	Hypothetical protein
VV1_1966	-1.506	Cytochrome c-type biogenesis protein CcmF
VV1_1967	-1.409	Thiol:disulfide interchange protein DsbE
VV1_1968	-1.557	Cytochrome c-type biogenesis protein CcmH
VV1_1969	-1.426	Cytochrome c-type biogenesis factor
VV1_2015	-2.909	Conserved hypothetical protein
VV1_2016	-2.662	Putative lactoylglutathione lyase
VV1_2220	-1.743	Phosphate acetyltransferase
VV1_2257	-1.344	6-pyruvoyl tetrahydrobiopterin synthase
VV1_2258	-1.581	Putative acetyltransferase
VV1_2259	-2.878	Hypothetical protein
VV1_2306	-1.328	Hypothetical protein
VV1_2308	-1.976	Putative glycosyltransferase protein
VV1_2309	-1.405	Putative glycosyltransferase protein
VV1_2311	-1.498	Hypothetical protein
VV1_2312	-1.351	Hypothetical protein
VV1_2388	-1.875	Hypothetical protein
VV1_2587	-1.622	Ferredoxin
VV1_2588	-1.748	Putative formate dehydrogenase-specific chaperone
VV1_2589	-1.955	Hypothetical protein
VV1_2591	-1.379	Formate dehydrogenase, iron-sulfur subunit
VV1_2891	-1.472	Hypothetical protien
VV1_2892	-2.391	Outer membrane protein

VV1_3006	-1.423	3-oxoacyl-(acyl-carrier-protein) synthase II
VV1_3007	-1.705	Hypothetical protein
VV1_3009	-1.577	3-oxoacyl-(acyl-carrier-protein) reductase
VV1_3010	-2.234	Malonyl CoA-acyl carrier protein transacylase
VV1_3011	-1.700	3-oxoacyl-(acyl-carrier-protein) synthase III
VV1_3108	-1.618	Hypothetical protein
VV1_3213	-2.033	Hypothetical protein
VV1_3214	-1.527	Hypothetical protein
VV1_3215	-1.795	Hypothetical protein
VV1_3217	-1.846	Hypothetical protein
VV1_3231	-1.380	Hypothetical protein
VV1_3234	-2.301	Hypothetical protein
VV2_0005	-2.076	Phosphoenolpyruvate synthase
VV2_0006	-1.575	Conserved hypothetical protein
VV2_0009	-1.865	Hypothetical protein
VV2_0010	-1.719	Anaerobic glycerol-3-phosphate dehydrogenase, subunit A
VV2_0011	-1.681	Anaerobic glycerol-3-phosphate dehydrogenase, subunit B
VV2_0019	-2.548	Alcohol dehydrogenase, class IV
VV2_0084	-1.978	FlpFap pilin component containing protein
VV2_0148	-1.620	Acetate kinase 2
VV2_0186	-1.608	Glycine cleavage system P protein (pyridoxal-binding)
VV2_0187	-2.155	Glycine cleavage system H protein (lipoate-binding)
VV2_0188	-2.932	Serine hydroxymethyltransferase
VV2_0376	-1.666	Putative sensor histidine kinaseresponse regulator
VV2_0477	-1.559	Methyl-accepting chemotaxis protein
VV2_0478	-2.491	Alanine racemase 2
VV2_0503	-3.842	Cold shock protein CspA
VV2_0519	-2.469	Cold shock protein CSD
VV2_0854	-2.417	Tryptophanase
VV2_0855	-1.495	Tryptophan-specific transport permease protein
VV2_1252	-1.532	1,4-alpha-glucan branching enzyme
VV2_1315	-1.323	Hypothetical protein
VV2_1318	-1.534	Pseudouridylate synthase, 23S RNA-specific
VV2_1355	-2.134	Putative integral membrane protein
VV2_1373	-1.704	Uridine phosphorylase
VV2_1484	-2.221	2-amino-3-ketobutyrate coenzyme A ligase
VV2_1485	-1.928	L-threonine 3-dehydrogenase
VV2_1543	-1.364	Deoxyribodipyrimidine photolyase
VV2_1690	-1.601	Hypothetical protein

[a] Locus tag numbers and annotation of gene products are based on the database of the *V. vulnificus* CMCP6 genome, which was retrieved from GenBank.

[b] The M value represents the $\log_2$ ratio of DNA hybridization intensity of each gene in the biofilms versus planktonic cells. The values shown are the mean from three independent experiments. The locus tags and genes with $M \geq 1.322$ or $M \leq -1.322$ (expression ratios of ≥ 2.5, $P < 0.05$) were considered as the genes differentially regulated in biofilms.
