# Supplementary material for: The cabABC Operon Essential for Biofilm and Rugose Colony Development in Vibrio vulnificus
Source: PLoS Pathog. 2015 Sep 25;11(9):e1005192. doi: 10.1371/journal.ppat.1005192 (PMC4584020; doi:10.1371/journal.ppat.1005192)
Supplement: S2 Table — (PDF) [file ppat.1005192.s005.pdf]

S2 Table. Preferential expression of *cabABC* in biofilms[a]

Locus tag	Gene	Product	Microarray		qRT-PCR	
			Mean Log_2 ratio[b]	*P*-value	Mean Log_2 ratio[b]	*P*-value
VV2_1571	*cabA*	A protein with putative calcium-binding motifs	2.766	6.67E-03	2.967	3.76E-04
VV2_1572	*cabB*	ABC-type transporter	1.544	2.50E-02	1.975	7.75E-07
VV2_1573	*cabC*	Membrane fusion protein (MFP)	1.411	2.92E-03	1.592	5.40E-04

[a] The consecutive *cabABC* genes were selected from the pool of genes that are up-regulated in biofilms (S1 Table). Their preferential expression in the biofilms was confirmed by qRT-PCR using the primer sets listed in S3 Table. Locus tag numbers and annotation of gene products are based on the database of the *V. vulnificus* CMCP6 genome, which was retrieved from GenBank. ABC-type transporter, ATP-binding cassette type transporter.

[b] The values represent the log_2 ratio of mRNA expression of each gene in the biofilms versus planktonic cells. The values shown are the mean from three independent experiments.
