# Supplementary material for: The cabABC Operon Essential for Biofilm and Rugose Colony Development in Vibrio vulnificus
Source: PLoS Pathog. 2015 Sep 25;11(9):e1005192. doi: 10.1371/journal.ppat.1005192 (PMC4584020; doi:10.1371/journal.ppat.1005192)
Supplement: S3 Table — (PDF) [file ppat.1005192.s006.pdf]

S3 Table. Oligonucleotides used in this study

Oligonucleotide[a]	Sequence (5' → 3')[b]	Use
RTcabA	CAGATTTTGTCGCTCATGCGG	RT-PCR of *cabABC*
RTcabC2	AGATAGCTGATTGGCGTCCGTG	
cabAexp_F	CCATGGCTGTTTATTCTGGAACTG	Amplification of the *cabA* coding region
cabAexp_R	CTCGAGGAATTGGAACATGTCATC	
dcpA_F1	ACCCCGCTTGTGAGGTGAAC	Integration of P_{BAD} with *dcpA*
dcpA_R1	GATTCCATGGCGTTAGCCAACTGTAAT	
dcpA_F2	AACGCCATGGAATCATATTTAGGTAAAGGAAG	
dcpA_R2	GAGCCCGTTAGATCAGAGACATGTCAG	
pBAD24_F	CCATGGTGCATAATGTGCCTGTC	
pBAD24_R	CCGGGTACCATGGTGAATTCCT	
cabA_F1	ACTAGTGGTGGAGCGAAGAAGGAA	Construction of the *cabA* mutant
cabA_R1	CGGGATCCGTTCCAGAATAAACA	
cabA_F2	ACGGATCCCGGATGACATGTT	
cabA_R2	GCATGCGATAAAGAGAGGAACCAC	
cabB_F1	GAAGTTGATTGGTGTGACAGAAGCAG	Construction of the *cabB* mutant
cabB_R1	AAGGATCCAAGCGTCTGTAAATCTCTC	
cabB_F2	TTGGATCCTTGCTCATA ATCCGAG	
cabB_R2	GACGGCATGGACAAACCGTC	
cabC_F1	AATAACGGAGAGAAATGGTGAGACGTTAG	Construction of the *cabC* mutant
cabC_R1	ATGGATCCTTCCCAGACAATCTCACT	
cabC_F2	AAGGATCCATGACCAAGATGCTCAG	
cabC_R2	GTACGTTTTCGACATCCGATATGATGAC	
brpA_F1	GCGGGTTTTAC GCCTATCGTGG	Construction of the *brpA* mutant
brpA_R1	CATCGGATCCATGTGCACATCTCGT	
brpA_F2	ACATGGATCCGATGCAGCTCATTG	
brpA_R2	GCAACTGTTGTTGATATTTCGCGAGGC	
cabAcom_F	AGTCCATGGTCCAATAATGGAATCAAAAGAGG	Complementation of the *cabA* mutant
cabAcom_R	CTGCATGCCCACCTAGAAGTAGTG	
cabBcom_F	AGCCATGGGTAGTAATT TGCAAAATTC	Complementation of the *cabB* mutant
cabBcom_R	AAGCATGCAACGTCTCACCATTTC	
cabCcom_F	AGCCATGGCTAATCAAATAACGGAG	Complementation of the *cabC* mutant
cabCcom_R	GAGCATGCATACATCAGTAATCATTCG	
qRTcabA_F	TTGGTTGCTGGCTCTGGTGAC	qRT-PCR
qRTcabA_R	ACTGTCTATACGCACTGTGTCCTC	
qRTcabB_F	GCCATTGCCCAGACCCAGAG	

qRTcabB_R	CCGATAATACCAACCGCACAACC
qRTcabC_F	TTGGCGGTGGTATTGGCTACTG
qRTcabC_R	TGTTGAATTGCCTGGCGTTGAC

[a] The oligonucleotides were designed using the *V. vulnificus* CMCP6 genome sequence.

[b] Regions of oligonucleotides not complementary to corresponding genes are underlined.
